# Learning Latent Trajectories in Developmental Time Series with Hidden-Markov Optimal Transport

**DOI:** 10.1101/2025.02.14.638351

**Published:** 2025-02-19

**Authors:** Peter Halmos, Julian Gold, Xinhao Liu, Benjamin J. Raphael

**Affiliations:** 1 Department of Computer Science, Princeton University, 35 Olden St, Princeton, NJ 08544; 2 Center for Statistics and Machine Learning, Princeton University, 26 Prospect Ave, Princeton, NJ 08544

## Abstract

Deriving the sequence of transitions between cell types, or differentiation events, that occur during organismal development is one of the fundamental challenges in developmental biology. Single-cell and spatial sequencing of samples from different developmental timepoints provide data to investigate differentiation but inferring a sequence of differentiation events requires: (1) finding trajectories, or ancestor:descendant relationships, between cells from consecutive timepoints; (2) coarse-graining these trajectories into a *differentiation map,* or collection of transitions between *cell types,* rather than individual cells. We introduce Hidden-Markov Optimal Transport (HM-OT), an algorithm that simultaneously groups cells into cell types and learns transitions between these cell types from developmental transcriptomics time series. HM-OT uses low-rank optimal transport to simultaneously align samples in a time series and learn a sequence of clusterings and a differentiation map with minimal total transport cost. We assume that the law governing cell-type trajectories is characterized by the joint law on consecutive time points, tantamount to a Markov assumption on these latent trajectories. HM-OT can learn these clusterings in a fully unsupervised manner or can generate the least-cost cell type differentiation map consistent with a given set of cell type labels. We validate the unsupervised clusters and cell type differentiation map output by HM-OT on a Stereo-seq dataset of zebrafish development, and we demonstrate the scalability of HM-OT to a massive Stereo-seq dataset of mouse embryonic development.

## 1 Introduction

**Figure 1:**
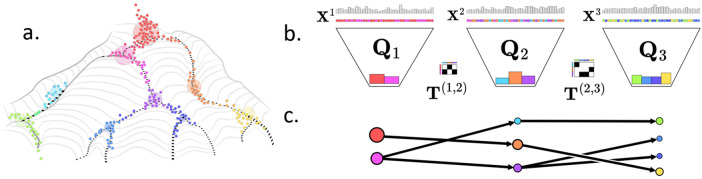
(**a**) Cells (colored points) occupy cell states on a Waddington landscape with undifferentiated cells having high differentiation potential at the top and fully differentiated cells with low differentiation potental at the bottom. Cells cluster into a small number of cell types (large circles). (**b**) Latent representation $Q_{t}$ is a joint distribution between cells $X^{1}$, $X^{2}$, $X^{3}$ at times 1, 2, 3, (top) and their cell types (bottom). Couplings $T^{\left(t,t+1\right)}$ describe cell type transitions across time. (**c**) A differentiation map summarizes the inferred latent trajectories with vertices indicating cell types and edges indicating transitions.

One of the most fundamental questions in biology is how a single zygote cell differentiates into the plethora of functionally specialized and distinct cell types, tissues, and organs which comprise a eukaryotic organism. In the past decades, a great deal of experimental work has elucidated the underlying mechanisms of development, a remarkably conserved process across all animals. From drosophila to humans, development is driven by cascading and spatially-segregated waves of epigenetic and transcriptomic changes. These changes are often summarized in a Waddington landscape [38] which describes cell types or cell states as a location on a landscape (often illustrated in 2D) and with each cell having a developmental potential, or height, describing its differentiation status. Multipotent progenitor cells have high developmental potential and roll down the landscape into differentiated cell types with lower developmental potential (Fig. 1). The key challenge in developmental biology can thus be phrased as learning the Waddington landscape of an organism including (1) the cell types and cell states; (2) a cell differentiation map that describes the developmental trajectories of cells on Waddington’s landscape.

Over the past few years, many researchers have attempted to derive developmental trajectories and differentiation maps from single-cell or spatial RNA sequencing data obtained from multiple time points during a developmental process. Since these technologies are destructive (an individual cell can be measured only once), the major problem in using this data is to link cells from these multiple time points into trajectories. This problem is usually termed *trajectory inference* and dozens of methods have been developed to solve this problem, with many of the earlier methods benchmarked in a DREAM challenge [28]. A key assumption in most of these methods is that the majority of cells occupy distinct cell types. These methods place cells on a small number of trajectories that connect these cell types, computing a continuous pseudotime for a cell along the trajectory. Other methods use heuristics that examine cells and cell types at consecutive time points. For example, [25, 26] construct cell differentiation maps by heuristics by connecting pairs of cell types from consecutive time points that have high proportions of nearest neighbors in a k-NN graph built from the cells at these time points.

Another popular approach for learning cell differentiation maps is to use the framework of *optimal transport* (OT) to learn couplings between two probability distributions over cell states. This approach was pioneered in Waddington-OT [33], a landmark paper and state of the art for single-cell alignment and trajectory inference. More recently, OT has extended to spatial transcriptomics data from multiple time points to learn trajectories in space and time [10, 17, 37]. These and other OT methods learn couplings, or alignments between pairs of *cells* from consecutive time points. Thus, deriving a differentiation map requires post-processing of the inferred couplings, with no guarantees that this coarse-graining is optimal or consistent across multiple time points.

We introduce *Hidden-Markov Optimal Transport (HM-OT),* an algorithm which takes as input a time-series of consecutive single-cell or spatial samples and uses low-rank OT [8, 30, 11] to infer both cell types at each timepoint *and* derive cell type transitions across timepoints. HM-OT leverages *Factor Relaxation with Latent Coupling (FRLC, mnemonic: “frolic”)* [11] – a new general-purpose algorithm for low-rank OT. FRLC solves for low-rank transport plans factored into three matrices: a pair of *latent representations* ($Q_{t}$, $Q_{t+1}$) and a *latent coupling (LC)* matrix ($T^{\left(t,t+1\right)}$) that links the two latent representations. HM-OT generalizes FRLC to a time-series of any length to derive consistent latent cell type representations (clusterings) across the whole time-series, overcoming limitations of pairwise methods that yield discordant cell type clusterings between time points. HM-OT optimizes a cost defined on trajectories through the time series which obeys an optimal sub-structure property. Owing to difficulties imposed by continuous dynamic programming, HM-OT approximates the MAP estimate instead, combining past and future information to learn latent representations and inferring couplings (cluster transitions) linking them into a sequence. The components returned by HM-OT define a joint distribution on the full time series. This joint distribution is coupled to a Markov chain on the latent (cell type) trajectories through the latent representations (cell type assignments). HM-OT is also flexible enough to learn a differentiation map *given* cell type annotations at each timepoint; we refer to this as “supervised HM-OT.”

We demonstrate the advantages of HM-OT on synthetic and real datasets, including a spatial RNA sequencing dataset of zebrafish embryogenesis from [21], and a spatial RNA-sequencing dataset of mouse embryogenesis [3]. Using information-theoretic metrics, we find that both HM-OT and supervised-HM-OT learn differentiation maps that are more biologically plausible than existing methods and accurately infer known cell type transitions.

## 2 Latent Trajectories and Optimal Transport

Given a time series $X=(X^{1},X^{2},\ldots ,X^{N})$ of cells – where each $X^{t}={\left\{x_{i}^{t}\right\}}_{i=1}^{n_{t}}$ are the cell states for a population of $n_{t}$ cells – our goal is to learn a collection of trajectories $x_{i}^{1}\to \cdots \to x_{j}^{t}\to \cdots \to x_{k}^{N}$ for each cell i that link cells from consecutive time points and describe the temporal evolution of individual cells over time. A widely used model in developmental biology to describe these trajectories is Waddington’s landscape [38, 33] which represents each cell state as a point on a landscape with multipotent cells having high developmental potential descending in key furrows of an epigenetic or transcriptomic landscape to a final differentiated population $X^{N}={\left\{x_{i}^{N}\right\}}_{i=1}^{n_{N}}$ (Fig. 1a).

Importantly, in organismal development cells group into a small number of cell states / cell types. Thus, computing trajectories of individual cells (i.e. a “full-rank” mapping from $X^{t}$ to $X^{t+1}$) is likely to overfit the data. Instead, the goal is to derive *latent trajectories* that connect cell types from populations of cells drawn from these cell types without prior knowledge of the cell types. We introduce the problem of discovering latent trajectories from a time series informally in Problem 1 below, which we will later formalize in Problem 2.

**Problem 1** (Latent trajectory inference problem, informal). *Suppose* $X={\left(X^{t}\right)}_{t=1}^{N}$ *is a time series with unknown trajectories* $\left(x_{i_{1}}^{1},\ldots ,x_{i_{N}}^{N}\right)$ *and latent trajectories* $\left(z_{k_{1}}^{1},\ldots ,z_{k_{N}}^{N}\right)$^1^. *Let* $Q_{t}$ *be the joint distribution between each* $x_{i_{t}}^{t}$ *and its latent state* $z_{k_{t}}^{t}$*, and assume* $z_{k_{t+1}}^{t+1}$ *is generated from* $z_{k_{t}}^{t}$*, under transition kernel* $\tilde{T}$. *Given* X*, find* Q *and* $\tilde{T}$.

As one concrete example, sampled single-cell trajectories $\left(x_{i_{1}}^{1},\ldots ,x_{i_{N}}^{N}\right)$ may be well-represented by only a handful of *discrete* latent trajectories $\left(z_{k_{1}}^{1},\ldots ,z_{k_{N}}^{N}\right)$, each such latent trajectory indexing a canal in the Waddington landscape. One can sample the initial “cell type” $Z^{1}∼P_{z^{1}}=\sum_{i}Q_{1}(x_{i}^{1},\cdot )$ and transition cell types according to transition kernel ${\tilde{T}}^{\left(t,t-1\right)}$ which generates $Z^{t}$ conditional on $Z^{t-1}$. Given the latent trajectory $\left(z_{k_{1}}^{1},z_{k_{2}}^{2},\ldots ,z_{k_{N}}^{N}\right)$, observations are sampled independently at each timepoint: $X^{t}∼P_{x^{t}|z^{t}}=Q_{t}\left(\cdot ,k_{t}\right)∕\sum_{i}Q_{t}(x_{i}^{t},k_{t})$. This generates an observed trajectory ($\left(x_{i_{1}}^{1},x_{i_{2}}^{2},\ldots ,x_{i_{N}}^{N}\right)$) and a latent trajectory ($\left(z_{k_{1}}^{1},\ldots ,z_{k_{N}}^{N}\right)$). Our focus on single-cell and spatial datasets motivates a formalization of Problem 1 using an empirical distribution over X and the framework of optimal transport to identify latent trajectories of *least-action* that are optimal with respect to this principle, which we describe below.

### 2.1 Optimal transport

Optimal transport (OT) is a framework for comparing and aligning datasets, encoded as probability distributions, using distances between the data points to determine the correct alignment. To describe this framework in general, suppose (X, d) is a metric space and let X, Y⊂X be two datasets, $X={\left\{x_{i}\right\}}_{i=1}^{n}$ and $Y={\left\{y_{j}\right\}}_{j=1}^{m}$. OT represents these datasets as discrete probability measures $\mu_{X}=\sum_{i=1}^{n}a_{i}\delta_{x_{i}}$ and $\mu_{Y}=\sum_{j=1}^{m}b_{j}\delta_{y_{j}}$, where $a\in \Delta_{n}$ and $b\in \Delta_{m}$ are probability vectors (often taken to be uniform), and $\Delta_{n}≔\{p\in R_{+}^{n}\;:\sum_{i}p_{i}=1\}$ denotes the probability simplex of size n. The goal of OT is to find a coupling matrix P with *marginals* $P1_{m}=a$, and $P^{T}1_{m}=b$ that minimizes a data-driven transport cost. Defining the sets $\Pi (a,\cdot )≔\{P\in R_{+}^{n\times m}\;:P1_{m}=a\}$, $\Pi (\cdot ,b)≔\{P\in R_{+}^{n\times m}\;:P^{T}1_{n}=b\}$, The set of *coupling matrices* or *transport plans* with marginals a and b is Π(a,b)=Π(a,⋅)∩Π(⋅,b). Given a cost $C\;:X\times X\to R_{+}$ and a cost matrix $c(x_{i},y_{j})={\left[C\right]}_{ij}\in R_{+}^{n\times m}$, the *Wasserstein problem* is to find $P^{⋆}\in \Pi (a,b)$ minimizing a transport cost over all coupling matrices:

(1) $$P^{⋆}≔{\text{argmin}}_{P\in \Pi (a,b)}{\langle P,C\rangle }_{F}=\sum_{i,j=1}^{n,m}P_{ij}C_{ij}.$$

The optimal transport cost itself, ${\langle P^{⋆},C\rangle }_{F}$ is called the *Wasserstein distance* between a and b. The Wasserstein problem finds a least-cost alignment between distributions. This has been applied in the seminal Waddington-OT [33] to find a least-cost alignment between single-cell transcriptomics distributions $P_{t}$ at different timepoints t.

### 2.2 Low-rank optimal transport with LC factorization

Low-rank OT [8, 30, 20, 32, 29] is a way of building interpretable cluster structure into OT by expressing transport plans as a product of low-rank factors performing an implicit clustering. Formally, low-rank OT constrains the *nonnegative rank* ${\text{rk}}_{+}(P)$ of coupling matrices P∈Π(a,b), defined as the smallest number of nonnegative rank-one matrices summing to P. For integer r≥1, let $\Pi_{r}(a,\cdot )≔\{P\in \Pi (a,\cdot )\;:{\text{rk}}_{+}(P)\leq r\}$, $\Pi_{r}(\cdot ,b)≔\{P\in \Pi (\cdot ,b)\;:{\text{rk}}_{+}(P)\leq r\}$, and define $\Pi_{r}(a,b)≔\Pi_{r}(a,\cdot )\cap \Pi_{r}(\cdot ,b)$. The low-rank balanced Wasserstein problem is (1) with $\Pi_{r}(a,b)$ in place of Π(a,b), and analogously for other marginal constraints and objectives. To solve low-rank OT problems, one must first choose a low-rank factorization of transport plans, with different clustering implications for each choice. Towards the construction of differentiation maps (where the number of cell types changes over time), we choose *latent coupling (LC) factorizations,* first introduced by [20] and recently extended to general costs by [11].

**Definition 1** (LC factorization). A latent coupling (LC) factorization *of a coupling matrix* $P\in \Pi_{r}(a,b)\subset R_{+}^{n\times m}$ *is a decomposition of the form*

(2) $$P=Q_{1}\text{diag}(1∕g_{1})T\text{diag}(1∕g_{2})Q_{2}^{T},$$

*where* $Q_{1}\in R_{+}^{n\times r_{1}}$, $Q_{2}\in R_{+}^{m\times r_{2}}$ *are the* latent representations *of* a *and* b*, respectively;* $T\in \Pi (g_{1},g_{2})\subset R_{+}^{r_{1}\times r_{2}}$ *is the* latent coupling; *and* $g_{1}≔Q_{1}^{T}1_{n}$ *and* $g_{2}≔Q_{2}^{T}1_{m}$ *are the* latent marginals.

One can imagine this decomposing an alignment between two datasets into (1) a map from a distribution over points in the first dataset (a) to their cluster distribution $\left(g_{1}\right)$ by $Q_{1}$, (2) a matrix of transitions T from the first cluster distribution to the second, and (3) a map from the second cluster distribution $\left(g_{2}\right)$ to a distribution over points in the second dataset (b) via $Q_{2}$ (Fig. 1b). In general, given a dataset $X={\left\{x_{i}\right\}}_{i=1}^{n}$ equipped with probability vector $a\in \Delta_{n}$, a *latent representation* of (X, a) is a coupling matrix $Q\in \Pi (a,\cdot )\subset R_{+}^{n\times r}$, where we typically suppose r≪n. Latent representations Q are joint distributions between points $x_{i}$ and r abstract latent points, and can be normalized to be row-stochastic or column-stochastic, with distinct interpretations. The row-stochastic normalization diag(1∕g)Q is a *soft clustering* of the dataset X using r labels, the column-stochastic normalization Λ≔Qdiag(1∕g) is a matrix of emission probabilities giving the likelihood of sampling data points in X conditional on one of the r labels, and column-stochastic $\tilde{T}=T\text{diag}(1∕g)$ is the cluster-cluster transition kernel.

Let ${\mathrm{LC}}_{a,b}(r_{1},r_{2})$ denote the set of LC factorizations ($Q_{1}$, $Q_{2}$, T) with $P_{\left(Q_{1},Q_{2},T\right)}\in \Pi_{a,b}$ and $T\in R_{+}^{r_{1}\times r_{2}}$, where $P_{\left(Q_{1},Q_{2},T\right)}$ is the product in Definition 1: $P_{\left(Q_{1},Q_{2},T\right)}≔Q_{1}\text{diag}(1∕g_{1})T\text{diag}(1∕g_{2})Q_{2}^{T}$. Using LC factorizations, the low-rank balanced Wasserstein problem is

(3) $$\underset{\left(Q_{1},Q_{2},T)\in {\mathrm{LC}}_{a,b}(r_{1},r_{2}\right)}{\text{min}}{\langle C,P_{\left(Q_{1},Q_{2},T\right)}\rangle }_{F},$$

which is equivalent to the objective optimized in [11].

## 3 Hidden Markov Optimal Transport

We extend the inference of latent transitions in the LC-factorization for pairwise optimal transport to N>2 timepoints. This motivates Problem 2 and Problem 3 below, which generalizes the approach to a time-series of any length N.

### 3.1 Temporal clustering using HM-OT

Below, we suppose that our time series $X={\left(X^{t}\right)}_{t=1}^{N}$ consists of datasets $X^{t}\subset X$ all lying in the same metric space (X, d), and write $X^{t}={\left(x_{i}^{t}\right)}_{i=1}^{n_{t}}$. For some cost function $c\;:X\times X\to R_{+}$, define cost matrices $C^{\left(t,t+1\right)}\in R_{+}^{n_{t}\times n_{t+1}}$ between consecutive time points via ${\left[C^{\left(t,t+1\right)}\right]}_{ij}≔c(x_{i}^{t},x_{j}^{t+1})$.

**Problem 2** (HM-OT problem). *Let* $X={\left(X^{t}\right)}_{t=1}^{N}$ *be a time series of* N *datasets, where each dataset* $X^{t}$ *is equipped with probability vector* $a_{t}\in \Delta_{t}$. *Given positive integers* $r_{1},\ldots ,r_{N}$, *and cost matrices* $C^{\left(t,t+1\right)}\in R_{+}^{n_{t}\times n_{t+1}}$, *find a sequence* $Q={\left(Q_{t}\in R_{t}^{n_{t}\times r_{t}}\right)}_{t=1}^{N}$ *of latent representations,* and *a sequence* $T={\left(T^{\left(t,t+1\right)}\in R_{+}^{r_{t}\times r_{t+1}}\right)}_{t=1}^{N-1}$ *of latent couplings that minimize:*

(4) $$\underset{Q,T\;:(Q_{1},Q_{t+1},T^{\left(t,t+1\right)})\in {\mathrm{LC}}_{a_{t},b_{t+1}}(r_{t},r_{t+1})}{\text{min}}\sum_{t=1}^{N-1}{\langle C^{\left(t,t+1\right)},P^{\left(t,t+1\right)}\rangle }_{F},$$

*where* $P^{\left(t,t+1\right)}≔Q_{t}\text{diag}(1∕g_{t})T^{\left(t,t+1\right)}\text{diag}(1∕g_{t+1})Q_{t+1}^{T}$.

Above, $P^{\left(t,t+1\right)}$ is defined as a special case of (6), with $g_{t}$ the latent marginal of $Q_{t}$, and $C^{\left(t,t+1\right)}$ are cost matrices between consecutive time points for cost function c. Note that the case that N=2, Problem 2 recovers the FRLC objective (3). As with FRLC, it is straightforward to generalize the total distance in the loss, replacing the Wasserstein cost in each term with GW or FGW (to handle spatial transcriptomics data). One can also relax the outer $\left(a_{t}\right)$ marginal constraints, as in [10], though we leave a thorough exploration of these relaxed constraints on our output to future work and focus on the balanced case, in which each $P^{\left(t,t+1\right)}\in \Pi (a_{t},a_{t+1})$. A solution to Problem 2 is a collection of latent representations $Q={\left(Q_{t}\right)}_{t=1}^{N}$ for the time series joined by a sequence of latent couplings $T={\left(T^{\left(t,t+1\right)}\right)}_{t=1}^{N-1}$; we call this sequence of latent couplings a *differentiation map* (Fig. 1c).

A differentiation map provides the minimum additional input required to describe a joint distribution across X, given a corresponding sequence of latent representations Q. In particular, we can express the joint distribution of a pair of timepoints s<t∈[N] which are not consecutive. First, define latent coupling matrix $T^{\left(s,t\right)}$ by

(5) $$T^{\left(s,t\right)}=T^{\left(s,s+1\right)}\text{diag}(1∕g_{s+1})T^{\left(s+1,s+2\right)}\text{diag}(1∕g_{s+2})\ldots \text{diag}(1∕g_{t-1})T^{\left(t-1,t\right)},$$

and define the joint distribution of timepoints s and t from Q, T as:

(6) $$P^{\left(s,t\right)}=Q_{s}\text{diag}(1∕g_{s})T^{\left(s,t\right)}\text{diag}(1∕g_{t})Q_{t}^{T}\;.$$

The factorization (5) is (up to the left-most factor) a composition of Markov transition kernels governing latent trajectories between times s and t. From the hidden-Markov perspective, column-stochastic normalizations Λ=Qdiag(1∕g) are matrices of emission probabilities. The interpretation of this structured coupling of timepoints $X^{s}$ and $X^{t}$ is: first, sample an entry of $T^{\left(s,t\right)}$, a pair of clusters, each at a distinct time point. Then, from each cluster, use the emission probabilities $\Lambda_{s}$, $\Lambda_{t}$ to sample an element of each timepoint. The emission matrices $\Lambda_{t}$ appear naturally in (6), so we will often write $P^{\left(s,t\right)}=\Lambda_{s}T^{\left(s,t\right)}\Lambda_{t}^{T}$ for brevity, with $T^{\left(s,t\right)}$ as in (5). We stress however that the matrices $Q={\left\{Q_{t}\right\}}_{t=1}^{N}$ and $T={\left\{T^{\left(t,t+1\right)}\right\}}_{t=1}^{N-1}$ are our variables of optimization in the algorithm presented below.

### 3.2 Computing the optimal Λ-sequence and approximate MAP estimate for HM-OT

We derive an algorithm to learn the set of latent representations and differentiation maps which minimize the objective 4 in HM-OT problem 2. First, we note that the loss in Problem 3 satisfies an optimal sub-structure property described below, where the optimal loss at time t depends only on the optimal loss at t−1. To see this, let θ=Λ, T and define

θt={Λ1ift=1(T(t−1,t),Λt)ift>1.}

Then the partial sums ${ℰ}_{t}(\theta_{t})$ express the minimization (4) recursively:

$${ℰ}_{t}(\theta_{t})≔\underset{\theta_{t-1}}{\text{min}}\left({\langle C^{\left(t-1,t\right)},\Lambda_{t-1}T^{\left(t-1,t\right)}\Lambda_{t}^{T}\rangle }_{F}+{ℰ}_{t-1}(\theta_{t-1})\right).$$

While dynamic programming (DP) principles have been applied to losses over sequences with similar optimal substructure properties, applying DP directly to Problem 2 has a number of serious limitations. In the context of HMMs, the Viterbi algorithm is used to infer the maximum-likelihood latent trajectory, under the assumption that the hidden state spaces are finite, and that emission probabilities and transition probabilities are known. In contrast, HM-OT learns both emission and transition probabilities, which themselves are the sequential variables. Moreover, the variables are real-valued, high-dimensional, and constrained, rendering the discretization necessary to apply a max-sum (Viterbi-type) DP infeasible. We choose instead to approximate the maximum a posteriori (MAP) estimate of $\Lambda_{t}$ using a message passing approach. This combines a forward-time “α-message” with a reverse-time message “β-message” to learn a consensus latent representation $\Lambda_{t}^{\gamma }$ from the full time series.

### 3.3 Forward, backward and consensus passes

The consensus γ-representation is conditional on the past (α) sequence $X^{1},\ldots ,X^{t-1}$ *and* the future (β) sequence $X^{t+1},\ldots ,X^{N}$. In S4.1.2, we derive Algorithm 1 to approximate the MAP estimate $\Lambda_{t}^{\gamma }={\text{argmax}}_{\Lambda_{t}}P_{\Lambda_{t}|{\left(X^{t}\right)}_{t=1}^{N}}$ which yields a least-cost clustering at each timepoint t, given all other time-points ${\left(X^{j}\right)}_{j\neq t}$. We note that this is distinct from finding the least-cost *sequence* $\left(\Lambda_{1}^{⋆},\ldots ,\Lambda_{N}^{⋆}\right)$ which a dynamic-programming approach would yield. Like the HMM or Kalman filter, the estimate of the MAP requires the defining a probability density (likelihood) over the variables of optimization. In our case, as we optimize a general energy function $ℰ\;:\Lambda_{i}\times T^{\left(i,i+1\right)}\to R$, this corresponds to a Boltzmann density on our energy

$$P_{\Lambda ,T}=\frac{1}{Z}\prod_{t=1}^{N-1}\psi_{t,t+1}≔\frac{1}{Z}\;\text{exp}\left\{-\sum_{t=1}^{N-1}{\langle C^{\left(t,t+1\right)},\Lambda_{t}T^{\left(t,t+1\right)}\Lambda_{t+1}^{T}\rangle }_{F}\right\},\;\;Z=\int_{\Lambda ,T}\prod_{t=1}^{N-1}\psi_{t,t+1}$$

where $\psi_{t,t+1}$ exponentiates each pairwise cost. We require a recursive set of integrals over future and past couplings to yield a marginal over each Λ-triple, $P_{\Lambda_{t-1,t,t+1}}$. We approximate each integral over the space of couplings by placing a δ-measure on the most-likely future and past couplings $\Lambda_{t-1}^{\alpha }$ and $\Lambda_{t+1}^{\beta }$, corresponding to the *ground-state* in each direction. This is the well-known *zero-temperature approximation,* where for temperature τ one takes the limit

$$\underset{\tau \to 0}{\text{lim}}P_{\theta }=\underset{\tau \to 0}{\text{lim}}\frac{1}{Z}e^{-ℰ\left(\theta \right)∕\tau }=\delta (\theta -\theta^{⋆}),\;\;\;\theta^{⋆}={\text{argmin}}_{\theta }ℰ(\theta )$$

Using this, we can channel information from the past and future sub-sequences with a pair of α, β-passes, avoiding an integration over the space of Λ, T. The α-pass (Algorithm 2) computes $\Lambda_{1:t-1}^{\alpha }$ as greedy forward-direction variables and the β-pass (Algorithm 3) $\Lambda_{t+1:N}^{\beta }$ as greedy backward-direction variables. Conditional on a forward message $\Lambda_{t-1}^{\alpha }$ and backward message $\Lambda_{t+1}^{\beta }$, we compute a smoothed variable $\Lambda_{t}^{\gamma }$ which marginally minimizes the summation objective 2 by minimizing a loss defined on $\Lambda_{t}^{\gamma }$ and costs over the local triple of timepoints t−1, t, t+1. The dependence on the full sequence $\left(X^{1},..,X^{\left(t-2\right)}\right)$, $\left(X^{\left(t+2\right)},..,X^{N}\right)$ efficiently factors through ($\Lambda_{t-1}^{\alpha }$, $\Lambda_{t+1}^{\beta }$) alone, allowing one to tractably compute MAP estimates for massive datasets $X^{\left(i\right)}$ over long sequence lengths N. As Algorithm 1 computes smoothed latent representations $\Lambda_{t}^{\gamma }$ as MAP estimates, the differentiation map is learned in a final completion step. The last step of Algorithm 1 computes optimal transitions between the latent representations to yield differentiation maps between the approximated MAP clusters. By the decoupling of the T matrices given Λ fixed, this constitutes a simple pairwise OT problem for $T^{\left(t,t+1\right)}\in R_{+}^{r_{t}\times r_{t+1}}$ given $\Lambda_{t}$, $\Lambda_{t+1}$. In fact, this last pass coincides with the fully-supervised variant of HM-OT, and we refer to (8) for the relevant optimization.

**Table:** 

Algorithm 1 HM-OT
$$\begin{array}{cc} \; & \;\mathrm{Require}:\text{Pairwise costs}\;{\left(C^{\left(n,n+1\right)}\right)}_{n=1}^{N-1}\\ \; & \;\;\;\text{Compute forward pass}\;Q_{1:N-1}^{\alpha }\leftarrow \alpha \text{-pass}(C^{\left(1,2\right)},\ldots ,C^{\left(N-1,N\right)})\;(\text{Algorithm}\;2)\\ \; & \;\;\;\text{Compute backward pass}\;Q_{2:N}^{\beta }\leftarrow \beta \text{-pass}(C^{\left(1,2\right)},\ldots ,C^{\left(N-1,N\right)})\;(\text{Algorithm}\;3)\\ \; & \;\;\;n\leftarrow N-1,m\leftarrow 1\\ \; & \;\;\;\mathrm{while}\;n\geq 2\;\mathrm{do}\\ \; & \;\;\;\;\;\;\;\Lambda_{n-1}^{\alpha }\leftarrow Q_{n-1}^{\alpha }\;\text{diag}(1∕g_{n-1}^{\alpha })\\ \; & \;\;\;\;\;\;\;\Lambda_{n+1}^{\beta }\leftarrow Q_{n+1}^{\beta }\;\text{diag}(1∕g_{n+1}^{\beta })\\ \; & \;\;\;\;\;\;\;\Lambda_{n}^{\gamma }\leftarrow {\text{argmin}}_{\Lambda_{n}^{\gamma }=Q_{n}^{\gamma }\;\text{diag}(1∕g_{n}^{\gamma }),T^{\left(n,n+1\right)}}{\langle C^{\left(n-1,n\right)},\Lambda_{n-1}^{\alpha }T^{\left(n-1,n\right)}\Lambda_{n}^{\gamma^{T}}\rangle }_{F}+{\langle C^{\left(n,n+1\right)},\Lambda_{n}^{\gamma }T^{\left(n,n+1\right)}\Lambda_{n+1}^{\beta ,T}\rangle }_{F}\\ \; & \;\;\;\;\;\;\;n\leftarrow n-1\\ \; & \;\;\;\mathrm{end}\;\mathrm{wile}\\ \; & \;\;\;\mathrm{while}\;m\leq N-1\;\mathrm{do}\\ \; & \;\;\;\;\;\;\;T_{\gamma }^{\left(n,n+1\right)}\leftarrow {\text{argmin}}_{T^{\left(n,n+1\right)}}{\langle C^{\left(n,n+1\right)},\Lambda_{n}^{\gamma }T^{\left(n,n+1\right)},\Lambda_{n+1}^{\gamma T}\rangle }_{F}\\ \; & \;\;\;\;\;\;\;m\leftarrow m+1\\ \; & \;\;\;\mathrm{end}\;\mathrm{while}\\ \; & \;\;\;\mathrm{return}\;\text{Smoothed clusters}\;\Lambda_{n}^{\gamma },\;\text{Transitions}\;T_{\gamma }^{\left(n,n+1\right)} \end{array}$$

### 3.4 Co-clustering the output of HM-OT

We introduce two approaches to cluster the time series X using HM-OT output. The first, *maximum-likelihood* co-clustering, finds the most likely clusters at each time-point and relates clusters across time-points through the latent coupling matrix. The second, *ancestral* co-clustering, factors the HM-OT output so that clusters are consistent across time. The former is valuable for identifying cell types and differentiation events. The latter is phylogenetic in nature, allowing one to trace cell types at any intermediate slice to their ancestors and descendants throughout the time series.

**Definition 2** (Maximum-likelihood clustering). *Given latent representations* ${\left\{Q_{t}\right\}}_{t=1}^{N}$, *a maximum-likelihood co-cluster* $\ell^{⋆}(i)$ *for a point* $i\in [n_{t}]$ *at time* t *is defined as the most-likely cluster assignment under* $Q_{t}$*:*

(7) $$\ell^{⋆}(i)={\text{argmax}}_{\ell \in [r_{t}]}P(x_{i}^{t},\ell )={\text{argmax}}_{\ell \in [r_{t}]}{\left[Q_{t}\right]}_{i,\ell }$$

*The clusters at times* t, t+1 *are put into (soft) correspondence by the cluster-cluster mapping* $T^{\left(t,t+1\right)}\;:R_{+}^{r_{t}}\to R_{+}^{r_{t+1}}$.

One can also cluster using a differentiation map by pulling back the mass of each descendant cell to its most likely ancestor, and using the latent cluster of the ancestor as its co-cluster. We call this *ancestral clustering,* defined in Section S5.2 and show one can co-cluster ancestor and descendant cells (Figure S2,S3). Unlike maximum-likelihood co-clustering, which depicts differentiation, ancestral co-clustering depicts “phylogenetic" relationships.

### 3.5 Learning differentiation maps from fixed cell type labels

In some applications, cluster labels for each sample are known; e.g. in a single-cell or spatial dataset, cell types may be annotated using marker genes or a reference dataset of cell types [3, 21, 39], leading to the Supervised HM-OT problem.

**Problem 3** (Supervised HM-OT problem). *The supervised HM-OT has an equivalent objective to 2, but with any subset* I⊂[N] *of the latent representations* ${(Q_{i})\;|}_{i\in I}$ *fixed, and with the* $T^{\left(i,i+1\right)}$ *and* ${(Q_{i})\;|}_{i\in [n]\setminus I}$ *remaining free-variables.*

We offer a solution to the Problem 3 and show in S2 how any input clustering at timepoint t has a unique latent representation $Q_{t}$. Thus, fixing cluster assignments for all t∈[N] uniquely determines all $Q_{t}$, hence $g_{t}$ and $\Lambda_{t}$. When all $Q_{t}$ variables are frozen according to cell type annotations, Problem 3 decouples in time and reduces to a sequence of *pairwise* OT problems for the optimal latent couplings $T^{\left(t,t+1\right)}$, as in the last step of Algorithm 1, reducing to:

(8) $$\sum_{t=1}^{N-1}\underset{T^{\left(t,t+1\right)}\in \Pi (g_{t},g_{t+1})}{\text{min}}{\langle T^{\left(t,t+1\right)},\Lambda_{t}^{T}C^{\left(t,t+1\right)}\Lambda_{t+1}\rangle }_{F},$$

noting the inner products in (8) are equivalent to those of (4). Thus, given annotated cell types as input, one may still find the differentiation map between them using HM-OT.

### 3.6 Evaluation metrics for differentiation maps

We adopt a widely-used metric to gauge the quality of a differentiation map $T={\left(T^{\left(t,t+1\right)}\right)}_{t=1}^{N-1}$.

#### Pointwise Mutual-Information (PMI) for Differentiation Maps.

Another measure of the quality of a differentiation map is the deviation between the probability of a transition between cell types a to b and the probability of the same transition under the independence model. Specifically, given the joint distribution $T^{\left(s,t\right)}$, defined in 5, and marginal distributions $g_{s}$, $g_{t}$ between any pair of timepoints s, t, we define the deviation using the *pointwise mutual information,* an information-theoretic metric widely used in machine-learning [15]:

$$\text{PMI}(a,b)=\log\frac{P_{s,t}(a,b)}{P_{s}(a)P_{t}(b)}=\log\frac{T_{a,b}^{\left(s,t\right)}}{{\left(g_{s}\right)}_{a}{\left(g_{t}\right)}_{b}}.$$

The normalized PMI (NPMI) scales PMI to be in [−1, +1]: where +1 indicates perfect correspondence between a and b, 0 indicates independence, and −1 indicates that a and b for never co-occur.

## 4 Experiments

### 4.1 Synthetic Experiments

We illustrate the advantages of HM-OT on two synthetic experiments (Fig. S1a,c) in Supplement S7.1. First, we demonstrate that HM-OT more accurately clusters data generated from an external field such as the Waddington gradient (Fig. S1a,b) outperforming k-means clustering applied independently to each time point (HM-OT adjusted mutual information AMI = 0.873, k-means AMI = 0.393). Second, we demonstrate the distinction between low-rank OT which co-clusters only 2 consecutive timepoints and HM-OT which simultaneously clusters trajectories with ≥ 2 timepoints (Fig. S1c,d). See SupplementS7.1.1,S7.1.2 for details.

### 4.2 Spatial-transcriptomics of Zebrafish development

**Figure 2:**
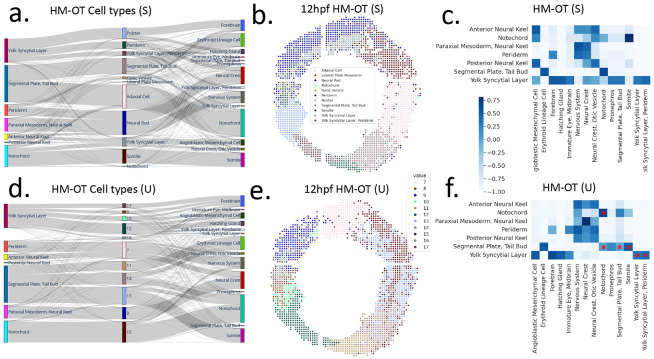
**(a)** Differentiation map output by HM-OT supervised with annotated 12hpf cell types [21], **(b)** Cell types for the 12hpf timepoint, **(c)** NPMI values between 10hpf and 18hpf for HM-OT supervised on annotated cell types. **(d)** Differentiation map output by HM-OT with unsupervised cell types, **(e)** cell types inferred by HM-OT for 12hpf without supervision, **(f)** NPMI values with HM-OT cell types. Stars indicate self-transitions and known biological transitions discussed in Section 4.2 .

We evaluate HM-OT’s ability to derive differntiation maps from spatial transcriptomics data by analyzing a dataset of zebrafish embryogenesis [21] that includes Stereo-Seq spatial transcriptomics data from multiple timepoints of embryonic zebrafish from stages 3.3hpf to 24hpf. We focus our analysis on three time points: 10hpf, 12hpf, and 18hpf. We first run HM-OT in supervised mode using the cell type annotations from [21], deriving a differentiation map shown in (Fig. 2a). Many of the transitions between cell types in this map do not agree with the known biology of zebrafish development including: notochord at 10hpf transitioning to somite at 12hpf; adaxial at 12hpf transitioning to notochord at 18hpf; and periderm transitioning to segmental plate/tail bud (Fig. 2a). Examining the spatial location of these annotated cell types (Fig. 2b), we observe that the notochord disappears at 12hpf (only 10 isolated spots out of 2081 total spots), but the notochord is a spatially coherent region at 10hpf and 18hpf (Fig. S6). This suggests that errors exist in the cell type labels, which may correspondingly affect the inferred map of cell type differentiation.

To further evaluate the differentiation map, we quantify the deviation of the observed trajectories from independence with the normalized pointwise mutual information (NPMI) value for cell types at 10hpf and 18hpf (Fig. 2c). We find that some well known transitions have low NPMI values (indicating that cell types are rarely on the same trajectory) such as notochord to notochord (NPMI = −0.107), tail-bud to notochord (NPMI = 0.033), and segmental plate to somite (NPMI = −0.965).

Because of the inconsistencies in the annotated cell types noted above, we investigate whether the unsupervised mode of HM-OT can infer better cell types (and consequently, a better differentiation map) on the 12hpf slice. We find that the differentiation map inferred by HM-OT unsupervised (Fig. 2d), is more biologically plausible than the differentiation map using the [21] annotated cell types. Specifically, HM-OT suggests notochord transitions to an intermediate cluster (unsupervised cluster 15 in Fig. 2d), which subsequently transitions to notochord, placing it on a single trajectory. In addition, this intermediate cluster 15 is spatially coherent and in the correct biological location (Fig. 2e). The notochord transition has a large NPMI value (NPMI = 0.665) as do several other known biological transitions ( Fig. 2f). For example, the transition from tail-bud to notochord, NPMI = 0.610, is also accurate [2]. Notably, HM-OT suggests the somite cell type arises from its correct progenitor, segmental plate and tail bud [34], with NPMI = 0.497. We offer all NPMI scores (Table S2,S1) and additional discussion of the differentiation maps, including errors common to both, in Supplement S7.2.3.

We further validated HM-OT unsupervised cluster 15 as the notochord cell type by examining differentially expressed genes. Our identified cluster 15 has N=333 spots and among the top 5 differentially expressed genes (T-test, Benjamini-Hochberg correction) are Chordin, a key notochord marker, (*chrd,* Z=6.15) and *ripply1* (Z=10.17) which is known to be found in the notochord [16]. In contrast, the top 5 differentially expressed genes for the notochord cell type at 12hpf using the annotations from [21] are *cirbpb* (Z=3.32, involved in cold-response), *phf5a* (Z=2.36, a splice factor), *rps3a* (Z=2.26, a ribosomal protein), *rpl39* (Z=2.17, a ribosomal protein), and *prpf4bb* (Z=2.09, an mRNA processing factor). None of these genes are known notochord markers.

For additional validation, we performed differential expression analysis on HM-OT clusters that are spatially adjacent to HM-OT cluster (15), which according to the annotations of [21] is a subset of the adaxial and somite cell type (N=438,265 spots). This region closely matches the region given by HM-OT clusters (13) and (15). In addition to (15) being a more likely candidate for the notochord cell type, we propose (13) as a better candidate for adaxial. The latter is suggested by the HM-OT differentiation map, placing (13) on a segmental plate - somite trajectory (Fig. 2d). In Supplement S7.2.2 we show that cell type 13 has muscular proteins characteristic of adaxial and that these proteins are much more weakly expressed in cell type 15. This result recapitulates developmental anatomy – the notochord is a rod-like structure which supports the neural tube and is surrounded by adaxial cells [24, 5]. Cell types 13 and 15 are spatially adjacent, with cell type 15 being more central, and cell type 13 flanking it on each side. Notably, the cell types of [21] relied on the use of a small number of marker genes, while the HM-OT cell types were inferred from whole-transcriptome expression. This suggests the unsupervised framework of HM-OT is able to infer cell types without requiring a panel of pre-annotated markers.

### 4.3 HM-OT differentiation map from a time series of whole-mouse embryogenesis

We demonstrate the scalability of HM-OT by analyzing the mouse organogenesis spatiotemporal transcriptomics atlas (MOSTA) dataset of [3]. This dataset contains spatial gene expression (Stereo-Seq platform) from entire mouse embryo at one day intervals between the time-stages of E9.5 to 16.5 with numbers of spatial locations ranging from 5,913 to 121,767. We compare the differentiation map computed by HM-OT with the map produced by moscot, a method for aligning pairs of spatial transcriptomics slices using optimal transport [17]. To our knowledge HM-OT and moscot are the only current methods that generate differentiation maps from spatiotemporal transcriptomics data and scale to the size of the MOSTA dataset. Both methods rely on low-rank optimal transport, so for both methods we set the rank of the transport matrix to be equal to the number of cell types between each aligned pair.

We find that HM-OT is both faster than moscot and also scales to larger datasets (Fig. S8a, Table S6). moscot runtimes (13.95s on N=5,913 spots for E9.5-10.5 to 25.65s on N=18,408 spots for E11.5-12.5) vary with dataset size (Fig. S8a,b), the runtime of HM-OT grows negligibly with the size of the dataset (8.98s for E9.5-10.5 to 9.31s on N=121,767 spots for E15.5-16.5). Moreover, moscot fails to run for alignments beyond the third pair of timepoints and fails to return a transport matrix past the second pair of timepoints (Fig. S8a). While moscot uses the low-rank factorization of $P=Q\text{diag}(1∕g)R^{T}$, this factorization does not include a latent transition matrix and the latent representations are not returned directly. Thus, one must return P in full to derive the differentiation map T. For the third pair of timepoints (E11.5 to E12.5) P is a matrix with 1.5-billion entries which exceeds GPU memory. In comparison, HM-OT computes the differentiation map without directly outputting P.

**Figure 3:**
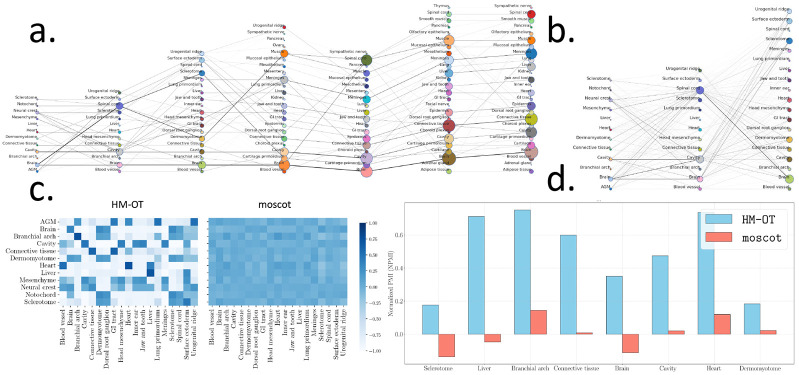
**a.** Differentiation map of mouse embryonic development derived by HM-OT with respect to the annotations of [3] across timepoints E9.5, E10.5, E11.5, E12.5, E13.5, E14.5, and E15.5. **b.** map of mouse differentiation inferred by moscot for E9.5, E10.5, and E11.5 (the map for E12.5 and above exceeded GPU memory). **c.** NPMI scores for all cell types for transitions inferred by HM-OT and moscot. **d.** Comparison on NPMI values for cell types which recur at E9.5 and E11.5 .

We evaluate the differentiation maps returned by HM-OT (Fig. 3a) and moscot(Fig. 3b), generating differentiation maps for both methods supervised by the cell type annotations in [3]. We quantify the difference between the two maps using the NPMI value defined in Section 3.6. We observe that HM-OT has a wide range of NMPI values with values either near +1 indicating that two cell types lie on a common trajectory or values near −1 indicating they will never share a trajectory (Fig. 3c). In contrast, moscot NPMI values are generally close to zero indicating near independence. Across the three timepoints (E9.5, E10.5, and E11.5) where both methods were able to run, HM-OT captures much stronger transitions between the same cell type than moscot. For all 8 cell types which recur from E9.5 to E11.5, HM-OT differentiation map has higher NPMI values than moscot(Fig. 3d). Moreover in the HM-OT map every cell type is substantially more linked to itself over time compared to moscot with a minimum NPMI = 0.176 ≫ 0. As an example, from E9.5 to E11.5 liver has NPMI = 0.713 (HM-OT) and NPMI = −0.0475 (moscot), branchial arch has NPMI = 0.751 (HM-OT) and NPMI = 0.144, heart has NPMI = 0.736 (HM-OT) and NPMI = 0.118 (moscot), and brain has NPMI = 0.3507 (HM-OT) and NPMI = −0.112 (moscot).

Beyond the E9.5-11.5 timepoint, HM-OT identifies well-known trajectories between differing cell types (Fig. 3a,c). For example, HM-OT identifies the urogenital ridge transitioning to ovary (NPMI = 0.560, E11.5-12.5) [35], the lung primordium transitioning to lung (NPMI = 0.927, E12.5-13.5) [23, 13], the dermomyotome transitioning to muscle (NPMI = 0.968, E11.5-12.5) [7], sclerotome transitioning to cartilage primordium (NPMI = 0.866, E11.5-12.5) [18], and surface ectoderm transitioning to the epidermis (NPMI = 0.705, E11.5-12.5) [27]. In cases where the cell type annotations are insufficiently resolved to distinguish local ectodermal versus mesodermal regions, one still finds connections between spatially co-localized and functionally related cell types such as the notochord and spinal cord as well as spinal cord and dorsal root ganglion [9]. In summary, HM-OT recapitulates known developmental trajectories in massive datasets, enabling the automated discovery of differentiation maps at once intractable scales.

Finally, to showcase the advantages of HM-OT, we visualize growth rates between E9.5-E15.5, derived as described in [10] (Fig. S7), the full map up to E16.5 (Fig. S4), and the co-clustering of mouse-embryonic dataset with both annotated and unsupervised clusters (Fig. S2).

## 5 Discussion

We introduce Hidden-Markov Optimal Transport (HM-OT) to learn latent temporal trajectories from transcriptomics data by simultaneously identifying latent representations of cells and transition matrices which link these representations across any number of timepoints. HM-OT can learn biologically plausible cell type differentiation maps, scaling to massive datasets out of reach for other methods. HM-OT has some limitations which are directions for future work. First, our algorithm computes an approximation of the maximum a posteriori (MAP) sequence of latent representations, and further investigation of scalable approaches to compute the optimal sequence are desirable. Second, our algorithm uses a non-convex objective and depends on hyper-parameters, most notably the number of cell types at each timepoint. Automated selection of these parameters and model selection are important subjects for future work. Finally, while we focused here on applications to spatiotemporal transcriptomics data, HM–OT is applicable to longitudinal single-cell transcriptomics data, and future work includes the evaluation of HM–OT on these data. We believe HM-OT and tools that build on it will enable mass-scale unsupervised discovery of the differentiation maps governing development.

## Supplementary Material

Supplement 1

## Footnotes

1 $i_{t}$ denotes an index into the point in $X^{t}$ on the trajectory, and $k_{t}$ denotes an index into the set of states $z_{k_{t}}$ can take
