# Supplementary material for: Learning Latent Trajectories in Developmental Time Series with Hidden-Markov Optimal Transport
Source: bioRxiv. 2025 Feb 19:2025.02.14.638351. Preprint. [Version 1] doi: 10.1101/2025.02.14.638351 (PMC11870411; doi:10.1101/2025.02.14.638351)
Supplement: Supplement 1 [file NIHPP2025.02.14.638351v1-supplement-1.pdf]

bioRxiv preprint doi: https://doi.org/10.1101/2025.02.14.638351; this version posted February 19, 2025. The copyright holder for this preprint (which was not certified by peer review) is the author/funder, who has granted bioRxiv a license to display the preprint in perpetuity. It is made available under aCC-BY 4.0 International license.

# Supplement

## S1 Optimal transport background

Let $(\mathsf{X}, \mathsf{d})$ be a metric space, with vector space structure, and let $\Delta_n = \{\boldsymbol{d} \in \mathbb{R}_+^n : \sum_{k=1}^n \boldsymbol{d}_k = 1\}$ be the set of probability vectors of size $n$. In computational optimal transport (OT), one encodes two datasets $\boldsymbol{X}, \boldsymbol{Y} \subset \mathsf{X}$,

$$\boldsymbol{X} = \{\mathbf{x}_1, \ldots, \mathbf{x}_n\}, \quad \boldsymbol{Y} = \{\mathbf{y}_1, \ldots, \mathbf{y}_m\}, \tag{9}$$

with $|\boldsymbol{X}| = n$, $|\boldsymbol{Y}| = m$ as discretely supported measures $\mu = \sum_{i=1}^n \boldsymbol{a}_i \delta_{\mathbf{x}_i}$ and $\nu = \sum_{j=1}^m \boldsymbol{b}_j \delta_{\mathbf{y}_j}$ which are represented by probability vectors $\boldsymbol{a} \in \Delta_n$ and $\boldsymbol{b} \in \Delta_m$. A joint density, or alignment, between $\mathbf{a}$ and $\mathbf{b}$ is called a *coupling*, which we define below.

**Definition 3** (Coupling matrix)**.** *The set of* coupling matrices *or* transport plans *between* $\boldsymbol{a}$ *and* $\boldsymbol{b}$ *is:*

$$\Pi(\boldsymbol{a}, \boldsymbol{b}) := \{\mathbf{P} \in \mathbb{R}_+^{n \times m} \; : \; \mathbf{P}\mathbf{1}_m = \boldsymbol{a}, \, \mathbf{P}^T\mathbf{1}_n = \boldsymbol{b}\}. \tag{10}$$

We call $\boldsymbol{a}$ and $\boldsymbol{b}$ the *left* and *right marginals* of $\mathbf{P} \in \Pi(\boldsymbol{a}, \boldsymbol{b})$. Using a *cost matrix* $\mathbf{C}$ derived from the data $\boldsymbol{X}, \boldsymbol{Y}$, one seeks a transport plan $\mathbf{P}^\star$ optimal with respect to an objective $\mathcal{H}$ which is a differentiable function of this alignment.

**Wasserstein distance (EMD)**

Given cost matrix $\mathbf{C} \in \mathbb{R}_+^{n \times m}$ where $\mathbf{C}_{ij} = c(\mathbf{x}_i, \mathbf{y}_j)$ for $c(\cdot, \cdot) : \mathsf{X} \times \mathsf{X} \to \mathbb{R}_+$, the *Wasserstein cost* of transport plan $\mathbf{P} \in \Pi(\boldsymbol{a}, \boldsymbol{b}) \subset \mathbb{R}_+^{n \times m}$ is $\mathcal{H}_{\mathrm{W}} := \langle \mathbf{P}, \mathbf{C} \rangle_F = \sum_{i=1}^n \sum_{j=1}^m \mathbf{P}_{ij} \mathbf{C}_{ij}$. The optimal value of the Wasserstein cost is the *Wasserstein distance between* $\boldsymbol{a}$ *and* $\boldsymbol{b}$ *with respect to* $\mathbf{C}$.

$$\mathrm{W}_{\mathbf{C}}(\boldsymbol{a}, \boldsymbol{b}) := \min_{\mathbf{P} \in \Pi(\boldsymbol{a}, \boldsymbol{b})} \langle \mathbf{P}, \mathbf{C} \rangle_F. \tag{11}$$

When $\mathsf{X}$ is Euclidean space $\mathbb{R}^d$, and when $c : \mathsf{X} \times \mathsf{X} \to \mathbb{R}$ is the squared-Euclidean distance, (11) defines the squared *2-Wasserstein distance* between $\boldsymbol{a}$ and $\boldsymbol{b}$. Wasserstein distance, in particular the 2-Wasserstein distance, is synonymous with *earth-mover's distance (EMD)*, as the objective value (11) can be interpreted as work or kinetic energy depending on the cost matrix $\mathbf{C}$. In typical biological applications, $\mathsf{X}$ is taken to be $\mathbb{R}^g$, and where $g$ denotes either the number of genes (the dimension of gene expression space) or the number of principal components of the expression vectors that are kept. In such applications, e.g. [33], cost function $c$ is usually chosen as the Euclidean distance in this expression space. While we specialize to the Euclidean distance in the present work, we note that all OT problems stated (including the `HM-OT` problem statement, Problem 2) extend to general costs, which `HM-OT` can handle.

**Gromov-Wasserstein and other objectives**

When datasets $\boldsymbol{X}, \boldsymbol{Y}$ have additional geometric structure (e.g. in spatial transcriptomics), the transport cost (1) can be augmented with a *Gromov-Wasserstein (GW)* term, using intra-dataset distance matrices $\mathbf{D}^{\boldsymbol{X}} \in \mathbb{R}_+^{n \times n}$ $\mathbf{D}^{\boldsymbol{Y}} \in \mathbb{R}_+^{m \times m}$ derived from the spatial coordinates of $\boldsymbol{X}, \boldsymbol{Y}$. The *Gromov-Wasserstein* cost of any $\mathbf{P} \in \Pi(\boldsymbol{a}, \boldsymbol{b})$, according to $\mathbf{D}^{\boldsymbol{X}}, \mathbf{D}^{\boldsymbol{Y}}$ is:

$$\mathrm{GW}_{\mathbf{D}^{\boldsymbol{X}}, \mathbf{D}^{\boldsymbol{Y}}}(\mathbf{P}) = \sum_{i,j,k,l} |[\mathbf{D}^{\boldsymbol{X}}]_{ij} - [\mathbf{D}^{\boldsymbol{Y}}]_{kl}|^2 \mathbf{P}_{ik} \mathbf{P}_{jl}. \tag{12}$$

This cost quantifies the metric distortion between $\boldsymbol{X}, \boldsymbol{Y}$ and the optimal $\mathbf{P}^\star = \mathrm{argmin}_{\mathbf{P} \in \Pi(\boldsymbol{a}, \boldsymbol{b})} \mathrm{GW}_{\mathbf{D}^{\boldsymbol{X}}, \mathbf{D}^{\boldsymbol{Y}}}(\mathbf{P})$ offers a geometric correspondence between $\boldsymbol{X}, \boldsymbol{Y}$. The *fused Gromov-Wasserstein (FGW) problem* finds a transport plan minimizing a convex combination of Wasserstein and Gromov-Wasserstein costs

$$\mathrm{argmin}_{\mathbf{P} \in \Pi(\boldsymbol{a}, \boldsymbol{b})} (1 - \alpha) \langle \mathbf{P}, \mathbf{C} \rangle_F + \alpha \mathrm{GW}_{\mathbf{D}^{\boldsymbol{X}}, \mathbf{D}^{\boldsymbol{Y}}}(\mathbf{P}). \tag{13}$$

Several works [40, 22, 17, 10] have used the FGW problem to align pairs of spatial transcriptomics datasets in different contexts. Other extensions, such as *semi-relaxed* and *unbalanced* OT, relax constraints on the marginals with a lower semi-continuous $\varphi$-divergence soft-penalty, such as KL. Such soft-penalties are more outlier-robust and can model growth and death. In particular, [33, 17] use unbalanced OT with curated marker genes to adjust the marginal $\boldsymbol{a}$ to account for growth and death over time, and [10] learns unsupervised growth rates using semi-relaxed OT.

It is possible that the two datasets $\boldsymbol{X} \subset \mathsf{X}, |\boldsymbol{X}| = n$ and $\boldsymbol{Y} \subset \boldsymbol{Y}, |\mathcal{Y}| = m$ live on distinct spaces, $\mathsf{X} \neq \mathsf{Y}$. Though we assume $\mathsf{X}$ and $\mathsf{Y}$ are each equipped with a metric, respectively $\mathsf{d}_{\mathsf{X}}$ and $\mathsf{d}_{\mathsf{Y}}$, there may not be a common metric $\mathsf{d}$ with

bioRxiv preprint doi: https://doi.org/10.1101/2025.02.14.638351; this version posted February 19, 2025. The copyright holder for this preprint (which was not certified by peer review) is the author/funder, who has granted bioRxiv a license to display the preprint in perpetuity. It is made available under aCC-BY 4.0 International license.

which to compare $x_i \in \mathcal{X}$ across the datasets to some $y_j \in \mathcal{Y}$. In this setting, we no longer have $\mathbf{C} \in \mathbb{R}_+^{n \times m}$. However, one can still form *intra-dataset distance matrices* $\mathbf{D}^{\mathcal{X}} \in \mathbb{R}_+^{n \times n}$ and $\mathbf{D}^{\mathcal{Y}} \in \mathbb{R}_+^{m \times m}$ from the data (9) via

$$[\mathbf{D}^{\mathcal{X}}]_{ij} := \mathsf{d}_{\mathsf{X}}(x_i, x_j), \quad [\mathbf{D}^{\mathcal{Y}}]_{k\ell} := \mathsf{d}_{\mathsf{Y}}(y_k, y_\ell). \tag{14}$$

These are used to define the *Gromov-Wasserstein objective*

$$\mathcal{H}_{\mathrm{GW}}(\mathbf{P}, \mathbf{D}^{\mathcal{X}}, \mathbf{D}^{\mathcal{Y}}) := \sum_{i,j,k,\ell} |\, [\mathbf{D}^{\mathcal{X}}]_{ij} - [\mathbf{D}^{\mathcal{Y}}]_{k\ell} \,|^2\, \mathbf{P}_{ik} \mathbf{P}_{j\ell}, \tag{15}$$

and the *Gromov-Wasserstein problem* for $\boldsymbol{a} \in \Delta_n$, $\boldsymbol{b} \in \Delta_m$ is the minimization of (15) over $\mathbf{P}$ with $\mathcal{H} = \mathcal{H}_{\mathrm{GW}}(\mathbf{P}, \mathbf{D}^{\mathcal{X}}, \mathbf{D}^{\mathcal{Y}})$. The minimum of the objective (15)

$$\mathrm{GW}(\boldsymbol{a}, \boldsymbol{b}) = \min_{\boldsymbol{P} \in \Pi(\boldsymbol{a}, \boldsymbol{b})} \sum_{i,j,k,\ell} |\, [\mathbf{D}^{\mathcal{X}}]_{ij} - [\mathbf{D}^{\mathcal{Y}}]_{k\ell} \,|^2\, \mathbf{P}_{ik} \mathbf{P}_{j\ell}$$

quantifies the minimum metric distortion incurred over all samples from $\mathbf{P}$, where each sample from $\mathbf{P}$ is an $(x, y)$-pair in $\mathcal{X} \times \mathcal{Y}$.

In other instances, one has access to both $\mathsf{d}$ and $\mathsf{d}_{\mathsf{X}}, \mathsf{d}_{\mathsf{Y}}$. In the simplest case, all three metrics coincide. However, it is possible that data $\mathcal{X}, \mathcal{Y}$ are additionally paired with accompanying features from different modalities, possibly distinct across the two datasets. In this case, with more structured data (and also in the simplest case), $\mathsf{d}_{\mathsf{X}}$ and $\mathsf{d}_{\mathsf{Y}}$ can be used to define intra-dataset distance matrices $\mathbf{D}^{\mathcal{X}}$, $\mathbf{D}^{\mathcal{Y}}$ for these modalities, as above in (14), and one can combine the Wasserstein and Gromov-Wasserstein objectives through a hyperparameter $\alpha \in (0, 1)$ [36]:

$$\mathcal{H}_{\mathrm{FGW}}(\mathbf{P}, \mathbf{C}, \mathbf{D}^{\mathcal{X}}, \mathbf{D}^{\mathcal{Y}}, \alpha) := (1 - \alpha)\langle \mathbf{P}, \mathbf{C} \rangle_F + \alpha \mathcal{H}_{\mathrm{GW}}(\mathbf{P}, \mathbf{D}^{\mathcal{X}}, \mathbf{D}^{\mathcal{Y}}). \tag{16}$$

Objective (16) is called the *fused Gromov-Wasserstein (FGW) objective*. FGW objectives have been used to align spatial transcriptomics data [40, 22, 17], and variants of FGW have been introduced [10] that include a sum over triples of points, effectively upweighting a subset of the terms in the sum (15).

## S2 Clustering and co-clustering

### Background on clustering

Let $(\mathsf{X}, \mathsf{d})$ be a metric space with vector space structure, and let $\mathcal{X} = \{x_1, \ldots, x_n\} \subset \mathsf{X}$ be finite a set. Suppose we are given a dissimilarity function $d : \mathsf{X} \times \mathsf{X} \to \mathbb{R}_+$ which satisfies reflexivity so that $d(x, x) = 0$, and symmetry so that $d(x, y) = d(y, x)\ \forall x, y \in \mathsf{X}$. Define $[\mathbf{C}]_{ij} := d(x_i, x_j)$ to be this dissimilarity function evaluated between all pairs of points in the finite set $\mathcal{X}$.

**Definition 4** (Clustering)**.** *Define $P(\mathcal{X})$ to be the set of all partitions of a set $\mathcal{X} = \{x_1, ..., x_n\}$. A* clustering function *on $\mathcal{X} \subset \mathsf{X}$ is a map $F : \mathbf{C} \to P(\mathcal{X})$. A* clustering *is the corresponding partition $F(\mathbf{C}) = \pi \in P(\mathcal{X})$ of the dataset.*

Generalizations of clustering often weight the points of $\mathcal{X}$ non-uniformly, corresponding to a weight vector $\mathbf{w}$ placed on the data points. In the context of optimal transport, we specialize to weights on the probability simplex $\mathbf{a} \in \Delta_{n-1} = \{\mathbf{a} : \sum_{i=1}^n \mathbf{a}_i = 1, \mathbf{a} \succeq \mathbf{0}\}$, and represent our dataset $\mathcal{X}$ as a measure supported on $\mathsf{X}$ weighted by $\mathbf{a}$, $\mu = \sum_{i=1}^n \mathbf{a}_i \delta_{x_i}$. For each clustering function $F$, one can define an additional probability vector $\mathbf{g}$, where $\mathbf{g}_i = \frac{|\mathcal{C}_i|}{|\pi|}$ for all clusters in the partition $\mathcal{C}_i \in \pi$. That is, the density $\mathbf{g}$ corresponds to (generally non-uniform) cluster proportions. Trivially, one can form a coupling $\gamma \in \Pi(\mathbf{a}, \mathbf{g})$ by defining an empirical joint measure $\sum_{i=1}^n \mathbf{a}_i \sum_{l=1}^{|\pi|} \delta_{q: x_i \in \mathcal{C}_q, l}$.

In a similar sense, we note that any map $F : \mathbf{C} \to P(\mathcal{X})$ can be equivalently represented by a matrix $\mathbf{Q} \in \{0, 1\}^{n \times |\pi|}$ where

$$\mathbf{Q}_{ij} = \begin{cases} 1 & \text{if} \quad i \in \mathcal{C}_j \\ 0 & \text{otherwise} \end{cases}$$

**Definition 5** (Soft clustering)**.** *A* soft clustering *is a relaxation of 6 where $\mathbf{Q}_{ij} \in \mathbb{R}_+$ and $\sum_j \mathbf{Q}_{ij} = 1$.*

As such, the definition of soft-clustering in 5 allows fuzzy assignments of points $i$ to clusters $j$ where the assignment may be split, e.g. between two clusters $j, j'$ which are both close to $i$.

bioRxiv preprint doi: https://doi.org/10.1101/2025.02.14.638351; this version posted February 19, 2025. The copyright holder for this preprint (which was not certified by peer review) is the author/funder, who has granted bioRxiv a license to display the preprint in perpetuity. It is made available under aCC-BY 4.0 International license.

**Definition 6** (Co-clustering)**.** *Define $P(\mathcal{X} \cup \mathcal{Y})$ to be the set of all partitions of the union of a set $\mathcal{X} = \{x_1, ..., x_n\}$ and a set $\mathcal{Y} = \{x_1, ..., x_m\}$. Let $[\mathbf{C}_{XY}]_{ij} = d(x_i, y_j)$ be a matrix with rows representing $\mathcal{X}$ and columns representing $\mathcal{Y}$. A* co-clustering function on $\mathcal{X} \times \mathcal{Y} \subset \mathsf{X} \times \mathsf{Y}$ *is a map $F : \mathbf{C}_{XY} \to P(\mathcal{X} \cup \mathcal{Y})$. A* co-clustering *is the corresponding partition $F(\mathbf{C}_{XY}) = \pi_{XY} \in P(\mathcal{X} \cup \mathcal{Y})$ of the row and column clusters of the matrix.*

As before, a co-cluster map $F$ can be represented in a matrix form – now with a pair of matrices $\mathbf{Q} \in \{0,1\}^{n\times|\pi|}$ and $\mathbf{R} \in \{0,1\}^{m\times|\pi|}$ where

$$\mathbf{Q}_{ij} = \begin{cases} 1 & \text{if} \quad x_i \in \mathcal{C}_j \\ 0 & \text{otherwise} \end{cases}$$

and

$$\mathbf{R}_{kj'} = \begin{cases} 1 & \text{if} \quad y_k \in \mathcal{C}_{j'} \\ 0 & \text{otherwise} \end{cases}$$

where $x_i$ and $y_k$ are said to be *co-clustered* if $j = j'$. In the case of co-clustering, one can observe that the cluster proportions allocated to $\mathcal{X}$ and $\mathcal{Y}$ may not be equivalent. In particular $|\{x \in \mathcal{X} : x \in \mathcal{C}_j\}| \neq |\{t \in \mathcal{Y} : y \in \mathcal{C}_j\}|$, and $n \neq m$ in general. Even more broadly, it may be so that the set of clusters $\mathcal{C}_j$ representing $\mathcal{X}$ and $\mathcal{Y}$ are jointly not as reasonable as a pair of sets of clusters $\mathcal{C}_j, \tilde{\mathcal{C}}_k$ allowed to have differing cluster-proportions allocated to $\mathcal{X}, \mathcal{Y}$ with a *joint-density* between the clusters themselves.

**Definition 7** (Joint-factored Co-clustering)**.** *Define $P(\mathcal{X}), P(\mathcal{Y})$ to be the set of all partitions of $\mathcal{X} = \{x_1, ..., x_n\}$ and $\mathcal{Y} = \{y_1, ..., y_m\}$. Let $[\mathbf{C}_{XY}]_{ij} = d(x_i, y_j)$ be a matrix with rows representing $\mathcal{X}$ and columns representing $\mathcal{Y}$. A* joint co-clustering function on $\mathcal{X} \times \mathcal{Y} \subset \mathsf{X} \times \mathsf{Y}$ *is a map $F : \mathbf{C}_{XY} \to P(\mathcal{X}), P(\mathcal{Y}), \mu_{XY}$ where $\mu_{XY}$ is a joint-density defined on the product-space of partitions $P(\mathcal{X}) \times P(\mathcal{Y})$.* Marginal clusterings *correspond to the partitions of $\mathcal{X}$ and $\mathcal{Y}$, $\pi_X$ and $\pi_Y$, and the* co-clustering *corresponds to partitions in $P(\mathcal{X} \cup \mathcal{Y})$ maximally-likely under the joint law.*

Under this definition, one allows both distinct clusters on $\mathcal{X}$ and $\mathcal{Y}$, as well as distinct cluster densities $(\boldsymbol{g}_Q)_j = \frac{|\mathcal{C}_j|}{n}$ and $(\boldsymbol{g}_R)_{j'} = \frac{|\tilde{\mathcal{C}}_{j'}|}{m}$ where the difference between clusters is mediated by the joint density, or coupling, in the set

$$\mu_{XY} \in \Pi\left(\sum_{i=1}^{|\pi_X|} \frac{|\mathcal{C}_j|}{n}\delta_{\mathcal{C}_j}, \sum_{j'=1}^{|\pi_Y|} \frac{|\tilde{\mathcal{C}}_{j'}|}{m}\delta_{\tilde{\mathcal{C}}_{j'}}\right)$$

with matrix-realization $\mathbf{T}_{jj'} = \mu_{XY}(\mathcal{C}_j, \tilde{\mathcal{C}}_{j'})$ quantifying the joint-density between clusters $j, j'$ of datasets $\mathcal{X}, \mathcal{Y}$. Having defined joint-factored co-clustering 7 under the assumption of hard 0-1 assignments, we note that there is a direct extension to soft co-clustering, analogous to 5, where the integral constraint is relaxed and one only requires that $\mathbf{Q}_{ij}, \mathbf{R}_{kj'} \in \mathbb{R}_+$ and $\sum_{j=1}^{|\pi_X|} \mathbf{Q}_{ij} = \sum_{j'=1}^{|\pi_Y|} \mathbf{R}_{kj'} = 1$.

**Connection to the LC-factorization**

One may encode any input clustering as a unique $\mathbf{Q}_i$ matrix of an LC-factorization, and given these $\mathbf{Q}_i$ matrices, can solve a sequence of *pairwise* OT problems for the differentiation map alone. Suppose one is given the cluster assignment of each sample $i$ to a cell type $l$ for times $t = 1, .., N$ as a map $F : [n_t] \to [r_t]$ for $r_t$ fixed cell types. Representing the encoding of cells by their type one can form the empirical joint measure, or coupling, given by $\gamma_t(\mu_n, \nu_r) = \frac{1}{n_t}\sum_{i=1}^{n_t}\sum_{\ell=1}^{r_t} \delta_{F(x_i),\ell}$ which has the matrix representation of $(\hat{\mathbf{Q}}_t)_{il} = \frac{\delta_{F(i),\ell}}{n_t}$. The row-sum represents the proportion of cluster $\ell$, $\sum_i(\hat{\mathbf{Q}}_t)_{i\ell} = \frac{\sum_{i:F(i)=\ell}}{n_t} = \frac{|\mathcal{C}_\ell|}{n_t}$ where $\mathcal{C}_\ell$ denotes the set of points assigned to cluster $\ell$. The column sum represents the proportion of spot $i$, taken to be $1/n_t$ for the empirical coupling, $\sum_\ell(\hat{\boldsymbol{Q}}_t)_{i\ell} = \frac{\sum_{\ell:F(i)=\ell}}{n_t} = \frac{1}{n_t}$. Thus $\hat{\mathbf{Q}}_t^T\mathbf{1}_{n_t} = \hat{\boldsymbol{g}}_{Q_t}$ has the cluster-distribution as its entries and $\hat{\mathbf{Q}}_t\mathbf{1}_{r_t} = \frac{1}{n_t}\mathbf{1}_{n_t}$ is uniform over points. Thus, having a cluster assignment uniquely determines an encoding of a sub-coupling matrix variable $\hat{\mathbf{Q}}_t$. Thus, given marginal clusterings $\pi_X, \pi_Y$ in 7, one uniquely determines two latent representations for $\mathbf{Q}_X, \mathbf{Q}_Y$, and the matrix realization of a joint-density in 7, $(\mathbf{T}_{XY})_{jj'} = \mu_{XY}(\mathcal{C}_j, \tilde{\mathcal{C}}_{j'})$, determines a latent coupling as in 1. Thus, a joint-factored co-clustering 7 uniquely determines an LC-parametrization $(\mathbf{Q}_X, \mathbf{Q}_Y, \mathbf{T}_{XY})$.

bioRxiv preprint doi: https://doi.org/10.1101/2025.02.14.638351; this version posted February 19, 2025. The copyright holder for this preprint (which was not certified by peer review) is the author/funder, who has granted bioRxiv a license to display the preprint in perpetuity. It is made available under aCC-BY 4.0 International license.

## S3 Temporal clustering

Temporal clustering has been explored in a number of works, such as Dey et al. [6]. We offer definitions of temporal clustering as it pertains to this work and offer a "generative" process by which one can sample trajectories from one's unlabeled dataset – e.g. having solved for $(\mathbf{Q}, \mathbf{T})$ using `HM-OT` these may correspond to low-cost trajectories with respect to transcriptional distance.

**Definition 8** (Latent trajectory clustering)**.** *Suppose* $(\mathsf{X}, \mathsf{d})$ *is a metric space, and let* $\mathbf{X} = (\mathbf{X}^1, \ldots, \mathbf{X}^N)$ *be a sequence of* $N$ *finite subsets of* $\mathsf{X}$*, where* $|\mathbf{X}^t| = n_t$*. Let* $\mathbf{x} = (\mathbf{x}^1, \ldots, \mathbf{x}^N) \in \mathbf{X}$ *be any fixed trajectory, and let Suppose* $(\Sigma_t)_{t=1}^N$ *be finite alphabets with* $|\Sigma_i| = r_i$*. A* trajectory clustering *is an assignment of any observed trajectory* $\mathbf{x}$ *in a metric space* $\mathsf{X}$ *to a latent sequence*

$$\mathbf{x} \mapsto \mathbf{z} = (z_{k_1}, ..., z_{k_N}) \in \Sigma_1 \times ... \times \Sigma_N$$

*The discrete sequence* $\mathbf{z}$ *is the* latent trajectory *of* $\mathbf{x}$.

Under this definition, there are $\prod_{i=1}^N k_i$ possible latent-trajectory assignments to an observed sequence $\mathbf{x}$. In many cases, the set of possible trajectories is much smaller than this – in the case of Waddington's canalization, $\Sigma_i$ represents an alphabet of cell-states on a trajectory where single-cell trajectories may be regarded as sparse in the space $\Sigma_1 \times ... \times \Sigma_N$. A reasonable assumption to make is that $\mathbf{x}^i$ is independent of all $\mathbf{x}^{j\neq i}, \mathbf{z}^{j\neq i}$ given $\mathbf{z}_{k_i}^i$, akin to the assumption that on this trajectory $\mathbf{z}$, the marginal distribution of $\mathbf{x}^i$ depends only on a latent state at time $i$. Under this assumption, one has the following factorization of the joint-density

$$\mathbb{P}(\mathbf{x}, \mathbf{z}) = \mathbb{P}((\mathbf{x}^1, .., \mathbf{x}^N), (z_{k_1}^1, .., z_{k_N}^N))$$

$$= \mathbb{P}((\mathbf{x}^1, .., \mathbf{x}^N) \mid (z_{k_1}^1, .., z_{k_N}^N))\mathbb{P}(z_{k_1}^1, .., z_{k_N}^N)$$

$$= \left[\prod_{i=1}^N \mathbb{P}(\mathbf{x}^i \mid z_{k_i}^i)\right] \mathbb{P}(z_{k_1}^1, .., z_{k_N}^N)$$

In the case that the trajectories $\mathbf{z}$ exhibit undirected structure, one may factor the joint over $\mathbf{z}$ by clique where the joint of the trajectory $(z_{k_1}^1, .., z_{k_N}^N)$ decomposes into a joint over pairs

$$= \left[\prod_{i=1}^N \mathbb{P}(\mathbf{x}^i \mid z_{k_i}^i)\right] \prod_{i=1}^{N-1} \mathbb{P}(z_{k_i}^i, z_{k_{i+1}}^{i+1}) \tag{17}$$

With directed structure over $z_{k_i}^i$: $z_{k_1}^1 \to z_{k_2}^2 \to ... \to z_{k_N}^N$, the joint over the trajectory $\mathbf{z}$ factorizes as a Markov chain

$$= \left[\prod_{i=1}^N \mathbb{P}(\mathbf{x}^i \mid z_{k_i}^i)\right] \mathbb{P}(z_{k_1}^1) \prod_{i=2}^{N} \mathbb{P}(z_{k_i}^i \mid z_{k_{i-1}}^{i-1}) \tag{18}$$

In other words, under our model parameters $\mathbf{Q}, \mathbf{T}$, 17 the joint on $(\mathbf{x}, \mathbf{z})$ has realization given as

$$\left[\prod_{i=1}^N \mathbb{P}(\mathbf{x}^i \mid z_{k_i}^i)\right] \prod_{i=1}^{N-1} \mathbb{P}(z_{k_i}^i, z_{k_{i+1}}^{i+1}) = \left[\prod_{i=1}^N \frac{\mathbf{Q}(\mathbf{x}^i, z_{k_i}^i)}{\sum_p \mathbf{Q}_i(\mathbf{x}_p^i, z_i^{k_i})}\right] \prod_{i=1}^{N-1} \mathbf{T}_i(z_{k_i}^i, z_{k_{i+1}}^{i+1})$$

and under 18 the joint on $(\mathbf{x}, \mathbf{z})$ has realization

$$\left[\prod_{i=1}^N \mathbb{P}(\mathbf{x}^i \mid z_{k_i}^i)\right] \prod_{i=1}^{N-1} \mathbb{P}(z_{k_i}^i, z_{k_{i+1}}^{i+1}) = \left[\prod_{i=1}^N \frac{\mathbf{Q}(\mathbf{x}^i, z_{k_i}^i)}{\sum_p \mathbf{Q}_i(\mathbf{x}_p^i, z_i^{k_i})}\right] \boldsymbol{g}_0(z_{k_0}^0) \prod_{i=1}^{N-1} \tilde{\mathbf{T}}_i(z_{k_i}^i \mid z_{k_{i+1}}^{i+1})$$

for transition kernel $\tilde{\mathbf{T}} = \mathbf{T}^{(t+1,t)} \operatorname{diag}(1/\boldsymbol{g}_t)$. Under this process, one can "generate" latent trajectories within one's dataset as follows:

$$z_{k_1}^1 \sim \boldsymbol{g}_0$$

$$z_{k_t}^t \sim \tilde{\mathbf{T}}(\cdot, z_{k_{t-1}}^{t-1})$$

bioRxiv preprint doi: https://doi.org/10.1101/2025.02.14.638351; this version posted February 19, 2025. The copyright holder for this preprint (which was not certified by peer review) is the author/funder, who has granted bioRxiv a license to display the preprint in perpetuity. It is made available under aCC-BY 4.0 International license.

and from the sequence $(z_{k_1}^1, ..., z_{k_N}^N)$, sample a trajectory of points for all $t \in [N]$ as

$$i_t \sim \text{Categorical}\left(\frac{\mathbf{Q}_t(\cdot, z_k^t)}{\sum_i \mathbf{Q}_t(\mathbf{x}_i^t, z_k^t)}\right)$$

$$\mathbf{x}_{i_t} = \mathbf{X}_{i_t,\cdot}^t$$

where $\mathbf{X}^t \in \mathbb{R}^{n_t \times k}$ is a data matrix with $n_t$ points (e.g. single-cell transcript vectors) of dimension $k$. Moreover, the question of finding a sparse set of trajectories generating $\mathbf{X}$ is one of interest, especially in the context of single-cell transcriptomics.

**Problem 4** (Latent trajectory subset problem, informal)**.** *Suppose* $\mathbf{X} = (\mathbf{X}^t)_{t=1}^N$ *is a time series where* $(\mathbf{x}^t)_{t=1}^N \in \mathbf{X}^t$ *are associated to latent trajectory*

$$\mathbf{z} \in (z_{k_1}^1, ..., z_{k_N}^N) \in \Sigma_1 \times ... \times \Sigma_N$$

*Identify the subset of latent trajectories* $\Omega \subset \Sigma_N$ *such that* $\mathbf{x} \in \mathbf{X} \implies \mathbf{x} \mapsto \mathbf{z} \in \Omega$*. Analogously, given a measure* $\mu$ *on* $\mathbf{X}$ *and a trajectory assignment* $F : (\mathbf{X}^t)_{t=1}^N \to \Sigma_1 \times ... \times \Sigma_N$*, one seeks a subset* $\Omega \subset \Sigma_1 \times ... \times \Sigma_N$ *such that* $\mu\left(\{\mathbf{x} : F(\mathbf{x}) \in \Sigma_1 \times ... \times \Sigma_N \setminus \Omega\}\right) = 0$ *and* $\mu\left(F^{-1}(\mathbf{z}) : \mathbf{z} \in \Omega\right) > 0$ *for* $F^{-1}(\mathbf{z}) = \{\mathbf{x} \in \mathbf{X} : F(\mathbf{x}) = \mathbf{z}\}$*.*

For example, Problem 4 seeks a subset of latent trajectories, corresponding to a sequence of latent cell states, which describes the sparse canalization of Waddington's landscape.

## S4 Solving the Multi-marginal transportation problem

Let $\mathbf{X} = (\mathbf{X}^1, \ldots, \mathbf{X}^N)$ be a time series of length $N$, with $\mathbf{X}^t = \{\mathbf{x}_i^t\}_{i=1}^{n_t}$, and each $\mathbf{X}^t$ equipped with probability vector $\boldsymbol{a}_t \in \Delta_{n_t}$. Given couplings $\mathbf{P}^{(t-1,t)} \in \Pi(\boldsymbol{a}_{t-1}, \boldsymbol{a}_t)$ and $\mathbf{P}^{(t,t+1)} \in \Pi(\boldsymbol{a}_t, \boldsymbol{a}_{t+1})$, let us express their total transport cost across three consecutive timepoints $\mathbf{X}^{t-1}$, $\mathbf{X}^t$, $\mathbf{X}^{t+1}$ in terms of random variables $(X^{t-1}, X^t) \sim \mathbf{P}^{(t-1,t)}$ and $(\tilde{X}^t, \tilde{X}^{t+1}) \sim \mathbf{P}^{(t,t+1)}$:

$$\mathbb{E}_{\mathbf{P}^{(t-1,t)}}\|X^t - X^{t-1}\| + \mathbb{E}_{\mathbf{P}^{(t,t+1)}}\|\tilde{X}^{t+1} - \tilde{X}^t\| \tag{19}$$

When $\mathbf{P}^{(t-1,t)}$ and $\mathbf{P}^{(t,t+1)}$ have no rank constraints, the problem of minimizing (19) over $\mathbf{P}^{(t-1,t)}$ and $\mathbf{P}^{(t,t+1)}$ decouples into a sum of independent optimizations. However, we constrain the transport to pass through a *consistent* set of clusters at each timepoint: not only are $\mathbf{P}^{(t-1,t)}$ and $\mathbf{P}^{(t,t+1)}$ are low-rank, but we further require their LC factorizations to share a common factor $\boldsymbol{\Lambda}_t$. To see this, suppose the sets $\mathbf{Z}^{t-1} = \{\mathbf{z}_i^{t-1}\}_{i=1}^{r_{t-1}}$, $\mathbf{Z}^t = \{\mathbf{z}_i^t\}_{i=1}^{r_t}$, and $\mathbf{Z}^{t+1} = \{\mathbf{z}_i^{t+1}\}_{i=1}^{r_{t+1}}$ label the clusters at the three time points, and define the following *emission probabilities* from matrices $\boldsymbol{\Lambda}_{t-1}$, $\boldsymbol{\Lambda}_t$, and $\boldsymbol{\Lambda}_{t+1}$:

$$\mathbb{P}_{\boldsymbol{\Lambda}_{t-1}}(\mathbf{x}_i^{t-1} \mid \mathbf{z}_h^{t-1}) := [\boldsymbol{\Lambda}_{t-1}]_{i,h}\,,$$
$$\mathbb{P}_{\boldsymbol{\Lambda}_t}(\mathbf{x}_j^t \mid \mathbf{z}_\ell^t) := [\boldsymbol{\Lambda}_t]_{j,\ell}\,,$$
$$\mathbb{P}_{\boldsymbol{\Lambda}_{t+1}}(\mathbf{x}_k^{t+1} \mid \mathbf{z}_q^{t+1}) := [\boldsymbol{\Lambda}_{t+1}]_{k,q}$$

and from latent coupling matrices $\mathbf{T}^{(t-1,t)}$ and $\mathbf{T}^{(t,t+1)}$, define the joint distributions over consecutive pairs of cluster labels:

$$\mathbb{P}_{\mathbf{T}^{(t-1,t)}}(\mathbf{z}_h^{t-1}, \mathbf{z}_\ell^t) := [\mathbf{T}^{(t-1,t)}]_{h,\ell}\,,$$
$$\mathbb{P}_{\mathbf{T}^{(t,t+1)}}(\mathbf{z}_\ell^t, \mathbf{z}_q^{t+1}) := [\mathbf{T}^{(t,t+1)}]_{\ell,q}$$

from which, (19) can be expressed as follows:

$$= \sum_{i,j} \mathbb{P}_{\mathbf{P}^{(t-1,t)}}(\mathbf{x}_i^{t-1}, \mathbf{x}_j^t)\|\mathbf{x}_j^t - \mathbf{x}_i^{t-1}\| + \sum_{j,k} \mathbb{P}_{\mathbf{P}^{(t,t+1)}}(\mathbf{x}_j^t, \mathbf{x}_k^{t+1})\|\mathbf{x}_k^{t+1} - \mathbf{x}_j^t\|$$

$$= \sum_{i,j} \sum_{h=1}^{r_{t-1}} \sum_{\ell=1}^{r_t} \mathbb{P}_{\boldsymbol{\Lambda}_{t-1}}(\mathbf{x}_i^{t-1} \mid \mathbf{z}_h^{t-1}) \mathbb{P}_{\mathbf{T}^{(t-1,t)}}(\mathbf{z}_h^{t-1}, \mathbf{z}_\ell^t) \mathbb{P}_{\boldsymbol{\Lambda}_t}(\mathbf{x}_j^t \mid \mathbf{z}_\ell^t)\|\mathbf{x}_j^t - \mathbf{x}_i^{t-1}\|$$

$$+ \sum_{j,k} \sum_{\ell=1}^{r_t} \sum_{q=1}^{r_{t+1}} \mathbb{P}_{\boldsymbol{\Lambda}_t}(\mathbf{x}_j^t \mid \mathbf{z}_\ell^t) \mathbb{P}_{\mathbf{T}^{(t,t+1)}}(\mathbf{z}_\ell^t, \mathbf{z}_q^{t+1}) \mathbb{P}_{\boldsymbol{\Lambda}_{t+1}}(\mathbf{x}_k^{t+1} \mid \mathbf{z}_q^{t+1})\|\mathbf{x}_k^{t+1} - \mathbf{x}_j^t\|$$

bioRxiv preprint doi: https://doi.org/10.1101/2025.02.14.638351; this version posted February 19, 2025. The copyright holder for this preprint (which was not certified by peer review) is the author/funder, who has granted bioRxiv a license to display the preprint in perpetuity. It is made available under aCC-BY 4.0 International license.

We can further simplify by pulling the common factors of $\mathbb{P}_{\mathbf{\Lambda}_t}(\mathbf{x}_j^t \mid \mathbf{z}_\ell^t)$.

$$= \sum_{j=1}^{n_t} \sum_{\ell=1}^{r_t} \mathbb{P}_{\mathbf{\Lambda}_t}(\mathbf{x}_j^t \mid \mathbf{z}_\ell^t) \Big( \sum_{i=1}^{n_{t-1}} \sum_{h=1}^{r_{t-1}} \mathbb{P}_{\mathbf{\Lambda}_{t-1}}(\mathbf{x}_i^{t-1} \mid \mathbf{z}_h^{t-1}) \mathbb{P}_{\mathbf{T}^{(t-1,t)}}(\mathbf{z}_h^{t-1}, \mathbf{z}_\ell^t) \|\mathbf{x}_j^t - \mathbf{x}_i^{t-1}\| + \sum_{k=1}^{n_{t+1}} \sum_{q=1}^{r_{t+1}} \mathbb{P}_{\mathbf{T}^{(t,t+1)}}(\mathbf{z}_\ell^t, \mathbf{z}_q^{t+1}) \mathbb{P}_{\mathbf{\Lambda}_{t+1}}(\mathbf{x}_k^{t+1} \mid \mathbf{z}_q^{t+1}) \|\mathbf{x}_k^{t+1} - \mathbf{x}_j^t\| \Big)$$

Thus, under our constraints on $\mathbf{P}^1$ and $\mathbf{P}^{(2)}$, objective (19) reduces to

$$= \sum_{j=1}^{n_t} \sum_{\ell=1}^{r_t} [\mathbf{\Lambda}_t]_{j,\ell} \Big( \sum_{i=1}^{n_{t-1}} \sum_{h=1}^{r_{t-1}} [\mathbf{\Lambda}_{t-1}]_{i,h} [\mathbf{T}^{(t-1,t)}]_{h,\ell} \|\mathbf{x}_j^t - \mathbf{x}_i^{t-1}\| + \sum_{k=1}^{n_{t+1}} \sum_{q=1}^{r_{t+1}} [\mathbf{T}^{(t,t+1)}]_{\ell,q} [\mathbf{\Lambda}_{t+1}]_{k,q} \|\mathbf{x}_k^{t+1} - \mathbf{x}_j^t\| \Big),$$

which can be expressed concisely as:

$$= \langle \mathbf{\Lambda}_t, \mathbf{C}^{(t,t+1)} \mathbf{\Lambda}_{t+1} \mathbf{T}^{(t,t+1),T} + \mathbf{C}^{(t-1,t),T} \mathbf{\Lambda}_{t-1} \mathbf{T}^{(t-1,t)} \rangle_F.$$

Thus, subject to this low-rank decomposition, to find a clustering which minimizes the total Wasserstein distance traveled across timepoints, the inner clustering must minimize an inner product dependent on its left and right neighbors in the total sequence.

### S4.1 Optimal substructure and discretized approximations for the optimal $\Lambda$-sequence

Let $\mathbf{V} = \mathbf{\Lambda}, \mathbf{T}$ denote all optimization variables. Let us group these by timepoint, defining

$$\mathbf{V}_t = \begin{cases} \mathbf{\Lambda}_1 & \text{if } t = 1 \\ (\mathbf{T}^{(t-1,t)}, \mathbf{\Lambda}_t) & \text{if } t > 1 \end{cases}$$

One can express our full loss recursively:

$$\min_{\{\mathbf{\Lambda}_t\}, \{\mathbf{T}^{(t,t+1)}\}} \sum_{t=1}^{N-1} \langle \mathbf{C}^{(t,t+1)}, \mathbf{\Lambda}_t \mathbf{T}^{(t,t+1)} \mathbf{\Lambda}_{t+1}^T \rangle_F = \min_{\mathbf{V}_N} \mathcal{E}_N(\mathbf{V}_N)$$

where $\mathcal{E}_t : \mathbb{R}_+^{n_t \times r_t} \times \mathbb{R}_+^{r_{t-1} \times r_t} \to \mathbb{R}_+$ is defined for $t = 2, \ldots, N$ via:

$$\mathcal{E}_t(\mathbf{V}_t) := \min_{\mathbf{\Lambda}_{t-1}} \Big( \langle \mathbf{C}^{(t-1,t)}, \mathbf{\Lambda}_{t-1} \mathbf{T}^{(t-1,t)} \mathbf{\Lambda}_t^T \rangle + \min_{\mathbf{T}^{(t-2,t-1)}} \mathcal{E}_{t-1}(\mathbf{V}_{t-1}) \Big)$$
$$= \min_{\mathbf{V}_{t-1}} \Big( \langle \mathbf{C}^{(t-1,t)}, \mathbf{\Lambda}_{t-1} \mathbf{T}^{(t-1,t)} \mathbf{\Lambda}_t^T \rangle + \mathcal{E}_{t-1}(\mathbf{V}_{t-1}) \Big),$$

while in the "base" case of $t = 1$, define

$$\mathcal{E}_1(\mathbf{V}_1) := \langle \mathbf{C}^{(1,2)}, \mathbf{\Lambda}_1 \mathbf{T}^{(1,2)} \mathbf{\Lambda}_2^T \rangle.$$

This satisfies the optimal sub-structure property of problems typically solved using dynamic programming. In fact, maximizing a sum over a graph structure is solved by the max-sum DP, of which a sub-case of the algorithm specialized to HMMs is commonly known as the Viterbi algorithm. A number of difficulties make an immediate solution of this problem using standard tabular dynamic programming intractable. The matrices $\mathbf{\Lambda}_t \in \mathbb{R}_+^{n_t \times r_t}$ and $\mathbf{T}^{(t,t+1)} \in \mathbb{R}_+^{r_t \times r_{t+1}}$ are real-valued, positive matrices. Thus, solving this as a dynamic programming problem requires a discretization of the space which would suffer from the curse of dimensionality, incurring error exponential in the dimension. Moreover, this optimization is heavily constrained: $\forall t \in [1, .., T]$ the matrices $(\mathbf{\Lambda}_t, \mathbf{\Lambda}_{t+1}, \mathbf{T}^{(t,t+1)})$ must satisfy

$$\mathbf{\Lambda}_t^T \mathbf{g}_{Q_t} = \mathbf{1}_{r_{t+1}}$$
$$\mathbf{\Lambda}_t \mathbf{g}_{Q_{t+1}} = \mathbf{1}_{r_t}$$
$$\mathbf{T}^{(t,t+1)} \mathbf{1}_{r_{t+1}} = \boldsymbol{g}_{\boldsymbol{Q}_t}$$
$$\mathbf{T}^{(t,t+1),T} \mathbf{1}_{r_t} = \boldsymbol{g}_{\boldsymbol{Q}_{t+1}}$$

bioRxiv preprint doi: https://doi.org/10.1101/2025.02.14.638351; this version posted February 19, 2025. The copyright holder for this preprint (which was not certified by peer review) is the author/funder, who has granted bioRxiv a license to display the preprint in perpetuity. It is made available under aCC-BY 4.0 International license.

while optimized over all $\mathbf{g}_t, \mathbf{g}_{t+1} \in \Delta_{r_t}, \Delta_{r_{t+1}}$. Thus, the discretization of the space would be highly non-trivial. While the problem does satisfy optimal sub-structure by virtue of being a loss over a sequence, being both continuous and highly constrained makes the application of DP infeasible, necessitating approximate or greedy solutions.

#### S4.1.1 Background: decomposing the marginal into forward and backward densities

Our sequence of datasets has the structure of a graph $G = (V, E)$, with vertices given by the marginal clusterings $v_t = \{\mathbf{\Lambda}_t\} \in V$ and edges $(t, t+1) \in E$ between timepoints in sequence represented by the joint distribution $(\mathbf{T}^{(t,t+1)})_{t=1}^{N-1}$. For such graphs with sequential structure, message-passing is a common means of inferring variables which minimize a loss over the graph. We provide relevant background for this, following the presentation in [1]. Suppose one has an undirected chain of nodes $(\mathbf{z}_1, ..., \mathbf{z}_N)$. In such a chain one can decomposes the joint probability of $\mathbb{P}(\mathbf{z}_1, ..., \mathbf{z}_N)$ into cliques consist of pairs of points $\mathcal{C}_{i,i+1} = \{\mathbf{z}_i, \mathbf{z}_{i+1}\}$, such that the joint probability decomposes into a product of clique potentials $\psi : \mathcal{C}_{i,i+1} \to \mathbb{R}$ normalized by a partition function $Z$:

$$\mathbb{P}_{\mathbf{z}_1,...,\mathbf{z}_N} = \frac{1}{Z} \prod_{i=1}^{N-1} \psi_{i,i+1}(\mathbf{z}_i, \mathbf{z}_{i+1})$$

$$Z = \int_{\mathbf{z}_1,...,\mathbf{z}_N} \prod_{i=1}^{N-1} \psi_{i,i+1}(\mathbf{z}_i, \mathbf{z}_{i+1}) d\mathbf{z}_1 d\mathbf{z}_2 ... d\mathbf{z}_N$$

In message-passing, one assumes that the potentials $\psi_{i,i+1}$ are given. Using these, message-passing allows for efficient marginalization over all states to yield a distribution on any given node $\mathbf{z}_n$ or local connected sequence of nodes $\mathbf{z}_n, .., \mathbf{z}_{n+q}$. It is straightforward to observe that this marginalization is simple to perform from both ends by memoization of "messages" which are passed from both directions. In particular, one may observe

$$\mathbb{P}_{\mathbf{z}_n} = \frac{1}{Z} \int_{\mathbf{z}_{1 \le i \ne n \le N}} \mathbb{P}_{\mathbf{z}_1,...,\mathbf{z}_N} d\mathbf{z}_1 ... d\mathbf{z}_N = \frac{1}{Z} \int_{\mathbf{z}_{1 \le i \ne n \le N}} \prod_{i=1}^{N-1} \psi_{i,i+1}(\mathbf{z}_i, \mathbf{z}_{i+1}) d\mathbf{z}_1 ... d\mathbf{z}_N$$

$$= \frac{1}{Z} \int_{\mathbf{z}_{j \ne n, N, 1}} \left( \left( \int_{\mathbf{z}_1} \psi_{1,2}(\mathbf{z}_1, \mathbf{z}_2) d\mathbf{z}_1 \right) \prod_{i=2}^{N-2} \psi_{i,i+1}(\mathbf{z}_i, \mathbf{z}_{i+1}) \left( \int_{\mathbf{z}_N} \psi_{N-1,N}(\mathbf{z}_{N-1}, \mathbf{z}_N) d\mathbf{z}_N \right) \right)$$

observing that we can compute these integrals and store them as functions of $\mathbf{z}_2$ and $\mathbf{z}_{N-1}$, we renotate the forward and backward messages to be

$$\mu_\alpha(\mathbf{z}_2) = \left( \int_{\mathbf{z}_1} \psi_{1,2}(\mathbf{z}_1, \mathbf{z}_2) d\mathbf{z}_1 \right), \qquad \mu_\beta(\mathbf{z}_{N-1}) = \left( \int_{\mathbf{z}_N} \psi_{N-1,N}(\mathbf{z}_{N-1}, \mathbf{z}_N) d\mathbf{z}_N \right)$$

so

$$:= \frac{1}{Z} \int_{\mathbf{z}_{j \ne n, N, 1}} \left( \mu_\alpha(\mathbf{z}_2) \prod_{i=2}^{N-2} \psi_{i,i+1}(\mathbf{z}_i, \mathbf{z}_{i+1}) \mu_\beta(\mathbf{z}_{N-1}) \right)$$

and continue inductively up to node $n$

$$= \frac{1}{Z} \int_{\mathbf{z}_{j \ne n, N, N-1, 1, 2}} \left( \int_{\mathbf{z}_2} \mu_\alpha(\mathbf{z}_2) \psi_{2,3}(\mathbf{z}_2, \mathbf{z}_3) d\mathbf{z}_2 \right) \prod_{i=3}^{N-3} \psi_{i,i+1}(\mathbf{z}_i, \mathbf{z}_{i+1}) \left( \int_{\mathbf{z}_{N-1}} \psi_{N-2,N-1}(\mathbf{z}_{N-2}, \mathbf{z}_{N-1}) \mu_\beta(\mathbf{z}_{N-1}) \right)$$

$$= \frac{1}{Z} \int_{\mathbf{z}_{j \ne n, N, N-1, 1, 2}} \mu_\alpha(\mathbf{z}_3) \prod_{i=3}^{N-3} \psi_{i,i+1}(\mathbf{z}_i, \mathbf{z}_{i+1}) \mu_\beta(\mathbf{z}_{N-2}) = ... = \frac{\mu_\alpha(\mathbf{z}_n) \mu_\beta(\mathbf{z}_n)}{Z}$$

Thus, the marginal on any node $\mathbb{P}_{\mathbf{z}_n}$ in the graph can be computed using a forward message $\mu_\alpha$ and backward message $\mu_\beta$.

bioRxiv preprint doi: https://doi.org/10.1101/2025.02.14.638351; this version posted February 19, 2025. The copyright holder for this preprint (which was not certified by peer review) is the author/funder, who has granted bioRxiv a license to display the preprint in perpetuity. It is made available under aCC-BY 4.0 International license.

#### S4.1.2 Approximating the MAP estimate over triples

**Proposition 1.** *Supposing `HM-OT` energy function 2 has density given by the Boltzmann distribution*

$$\mathbb{P}_{\mathbf{\Lambda},\mathbf{T}} = \frac{1}{Z}\prod_{i=1}^{N-1}\psi_{i,i+1} := \frac{1}{Z}\exp\left\{-\sum_{t=1}^{N-1}\langle \mathbf{C}^{(t,t+1)}, \mathbf{\Lambda}_t \mathbf{T}^{(t,t+1)}\mathbf{\Lambda}_{t+1}^T\rangle_F\right\}, \qquad Z = \int_{\mathbf{\Lambda},\mathbf{T}}\prod_{i=1}^{N-1}\psi_{i,i+1}$$

*For pairwise clique energies, $\psi_{t,t+1} \propto \exp\left[-\langle \mathbf{C}^{(t,t+1)}, \mathbf{\Lambda}_t \mathbf{T}^{(t,t+1)}\mathbf{\Lambda}_{t+1}^T\rangle_F\right]$, the MAP estimate over triples $\mathbb{P}_{\mathbf{\Lambda}_{n-1},\mathbf{\Lambda}_n,\mathbf{\Lambda}_{n+1}}$ can be approximated by Algorithm 1 assuming a zero-temperature approximation on the density over $\psi_{n,n+1}$ in the recurrent integral*

$$\min_{\mathbf{\Lambda}_{n+1}} \log \int_{\Omega_n} \psi_{n+1,n}(\mathbf{\Lambda}_{n+1}, \mathbf{\Lambda}_n) d\mathbf{\Lambda}_n$$

*Proof.* Using the derivation of S4.1.1, one sees that the joint is expressed as

$$\mathbb{P}_{\mathbf{\Lambda}_{n-1},\mathbf{\Lambda}_n,\mathbf{\Lambda}_{n+1}} = \frac{1}{Z}\mu_\alpha(\mathbf{\Lambda}_{n-1})\psi_{n-1,n}(\mathbf{\Lambda}_{n-1},\mathbf{\Lambda}_n)\psi_{n,n+1}(\mathbf{\Lambda}_n,\mathbf{\Lambda}_{n+1})\mu_\beta(\mathbf{\Lambda}_{n+1})$$

and

$$\log \mathbb{P}_{\mathbf{\Lambda}_{n-1},\mathbf{\Lambda}_n,\mathbf{\Lambda}_{n+1}} = \log \mu_\alpha(\mathbf{\Lambda}_{n-1}) + \log \psi_{n-1,n}(\mathbf{\Lambda}_{n-1},\mathbf{\Lambda}_n) + \log \psi_{n,n+1}(\mathbf{\Lambda}_n,\mathbf{\Lambda}_{n+1}) + \log \mu_\beta(\mathbf{\Lambda}_{n+1}) - Z$$

The partition function, which normalizes the Boltzmann exponent, is

$$Z = \int_{\mathbf{\Lambda},\mathbf{T}}\prod_{i=1}^{N-1}\psi_{i,i+1}$$

and is constant with respect to $\mathbf{\Lambda}$ and $\mathbf{T}$. Thus, this reduces in proportionality to

$$\log \mathbb{P}(\mathbf{\Lambda}_{n-1},\mathbf{\Lambda}_n,\mathbf{\Lambda}_{n+1}) = \log \mu_\alpha(\mathbf{\Lambda}_{n-1}) + \log \psi_{n-1,n}(\mathbf{\Lambda}_{n-1},\mathbf{\Lambda}_n) + \log \psi_{n,n+1}(\mathbf{\Lambda}_n,\mathbf{\Lambda}_{n+1}) + \log \mu_\beta(\mathbf{\Lambda}_{n+1})$$

where, for simplicity, the dependence on the transition is left implicit

$$\log \psi_{n-1,n}(\mathbf{\Lambda}_{n-1},\mathbf{\Lambda}_n) := -\langle \mathbf{C}^{(n-1,n)}, \mathbf{\Lambda}_{n-1}\mathbf{T}^{(n-1,n)}\mathbf{\Lambda}_n\rangle_F$$

the first core approximation made is as follows

$$\max_{\mathbf{\Lambda}_{n-1},\mathbf{\Lambda}_n,\mathbf{\Lambda}_{n+1}} \log \mathbb{P}_{\mathbf{\Lambda}_{n-1},\mathbf{\Lambda}_n,\mathbf{\Lambda}_{n+1}} = \log \mu_\alpha(\mathbf{\Lambda}_{n-1}) + \log \psi_{n-1,n}(\mathbf{\Lambda}_{n-1},\mathbf{\Lambda}_n) + \log \psi_{n,n+1}(\mathbf{\Lambda}_n,\mathbf{\Lambda}_{n+1}) + \log \mu_\beta(\mathbf{\Lambda}_{n+1})$$

$$\geq \max_{\mathbf{\Lambda}_{n-1}} \log \mu_\alpha(\mathbf{\Lambda}_{n-1}) + \max_{\mathbf{\Lambda}_n}\left[\log \psi_{n-1,n}(\mathbf{\Lambda}_{n-1}^\alpha,\mathbf{\Lambda}_n) + \log \psi_{n,n+1}(\mathbf{\Lambda}_n,\mathbf{\Lambda}_{n+1}^\beta)\right] + \max_{\mathbf{\Lambda}_{n+1}} \log \mu_\beta(\mathbf{\Lambda}_{n+1})$$

where we maximize a lower-bound on the log-likelihood rather than the log-likelihood itself for

$$\mathbf{\Lambda}_{n-1}^\alpha = \mathrm{argmax}_{\mathbf{\Lambda}_{n-1}}\,\mu_\alpha(\mathbf{\Lambda}_{n-1}), \qquad \mathbf{\Lambda}_{n+1}^\beta = \mathrm{argmax}_{\mathbf{\Lambda}_{n+1}}\,\mu_\beta(\mathbf{\Lambda}_{n+1})$$

This involves first recursively maximizing a forward message $\mu_\alpha(\mathbf{\Lambda}_{n-1})$ and a backward message $\mu_\beta(\mathbf{\Lambda}_{n+1})$, fixing $\mathbf{\Lambda}_{n-1}^\alpha$ and $\mathbf{\Lambda}_{n+1}^\beta$ as the associated optimal arguments and then minimizing the $\mathbf{\Lambda}_n$ dependent term as

$$\min_{\mathbf{\Lambda}_n}\left[-\log \psi_{n-1,n}(\mathbf{\Lambda}_{n-1}^\alpha,\mathbf{\Lambda}_n) - \log \psi_{n,n+1}(\mathbf{\Lambda}_n,\mathbf{\Lambda}_{n+1}^\beta)\right]$$

$$= \min_{\mathbf{\Lambda}_n}\langle \mathbf{C}^{(n-1,n)}, \mathbf{\Lambda}_{n-1}^\alpha \mathbf{T}^{(n-1,n)}\mathbf{\Lambda}_n^T\rangle_F + \langle \mathbf{C}^{(n,n+1)}, \mathbf{\Lambda}_n \mathbf{T}^{(n,n+1)}\mathbf{\Lambda}_{n+1}^{\beta T}\rangle_F$$

To compute the recursive value for $\mu_\alpha$ requires an integration over a space of positive, stochastic matrices $\mathbf{\Lambda}_n \in \Omega_n$ subject to the structural constraint that

$$\mathbf{\Lambda}_n^T \mathbf{g}_{Q_i} = \mathbf{1}_{r_{i+1}}, \qquad \mathbf{\Lambda}_n \mathbf{g}_{Q_{i+1}} = \mathbf{1}_{r_i}, \qquad \mathbf{\Lambda}_n \succ \mathbf{0}$$

bioRxiv preprint doi: https://doi.org/10.1101/2025.02.14.638351; this version posted February 19, 2025. The copyright holder for this preprint (which was not certified by peer review) is the author/funder, who has granted bioRxiv a license to display the preprint in perpetuity. It is made available under aCC-BY 4.0 International license.

over all $\mathbf{g}_{Q_i}, \mathbf{g}_{Q_{i+1}} \in \Delta_{r_i}, \Delta_{r_{i+1}}$. The associated integral is then

$$\max_{\mathbf{\Lambda}_n} \log \mu_\alpha(\mathbf{\Lambda}_n) = \max_{\mathbf{\Lambda}_n} \log \int_{\Omega_{n-1}} \mu_\alpha(\mathbf{\Lambda}_{n-1}) \psi_{n,n-1}(\mathbf{\Lambda}_n, \mathbf{\Lambda}_{n-1}) d\mathbf{\Lambda}_{n-1}$$

$$= \max_{\mathbf{\Lambda}_n} \log \int_{\Omega_{n-1}} \mu_\alpha(\mathbf{\Lambda}_{n-1}) e^{-\langle \mathbf{C}^{(n,n-1)}, \mathbf{\Lambda}_{n-1} \mathbf{T}^{(n-1,n)} \mathbf{\Lambda}_n \rangle_F} d\mathbf{\Lambda}_{n-1}$$

To simplify this, consider the boundary case of $n = 1$ (resp. $n = N$):

$$\max_{\mathbf{\Lambda}_2} \log \mu_\alpha(\mathbf{\Lambda}_2) = \max_{\mathbf{\Lambda}_2} \log \int_{\Omega_1} \psi_{2,1}(\mathbf{\Lambda}_2, \mathbf{\Lambda}_1) d\mathbf{\Lambda}_1$$

We use the zero-temperature approximation where a Boltzmann distribution is approximated by a $\delta$-function on its ground-state

$$\frac{1}{Z} e^{-\mathcal{E}(\theta)} \approx \delta(\theta - \theta^*), \qquad \theta^* = \mathrm{argmin}_\theta\, \mathcal{E}(\theta)$$

so that annealing the Boltzmann distribution $\psi_{n,n-1}$ with a temperature parameter $\tau$, by taking $\tau \to 0$ we approximate the density with a point mass on the most likely configuration (ground-state)

$$\psi^\tau_{n,n-1} = \frac{1}{Z} \exp\left\{ -\frac{\langle \mathbf{C}^{(n-1,n)}, \mathbf{\Lambda}_{n-1} \mathbf{T}^{(n-1,n)} \mathbf{\Lambda}_n \rangle_F}{\tau} \right\}$$

$$\lim_{\tau \to 0} \psi^\tau_{n,n-1} = \delta(\mathbf{V} - \mathrm{argmin}_{\mathbf{V}} \langle \mathbf{C}^{(n-1,n)}, \mathbf{\Lambda}_{n-1} \mathbf{T}^{(n-1,n)} \mathbf{\Lambda}_n \rangle_F)$$

This replaces the intractable to integrate space of $\Omega_1$ with an integral on a Dirac delta-measure on the ground-state so that

$$\log \mu_\alpha(\mathbf{\Lambda}_2) := \log \int_{\Omega_1} \psi_{2,1}(\mathbf{\Lambda}_2, \mathbf{\Lambda}_1) \delta(\mathbf{\Lambda}_1 - \mathbf{\Lambda}_1^\alpha) d\mathbf{\Lambda}_1 = \log \psi_{2,1}(\mathbf{\Lambda}_2, \mathbf{\Lambda}_1^\alpha)$$

$$\mathbf{\Lambda}_2^\alpha = \mathrm{argmin}_{\mathbf{\Lambda}_2} \left[ -\log \psi_{2,1}(\mathbf{\Lambda}_2, \mathbf{\Lambda}_1^\alpha) \right]$$

This approximation allows us to compute the forward message $\mu_\alpha$ and with identical reasoning the backward message $\mu_\beta$. The recursion as expressed continues this approximation as

$$\log \mu_\alpha(\mathbf{\Lambda}_n) = \log \int_{\Omega_{n-1}} \psi_{n,n-1}(\mathbf{\Lambda}_n, \mathbf{\Lambda}_{n-1}) \mu_\alpha(\mathbf{\Lambda}_{n-1}) d\mathbf{\Lambda}_{n-1} = \log \psi_{n,n-1}(\mathbf{\Lambda}_n, \mathbf{\Lambda}^\alpha_{n-1}) \mu_\alpha(\mathbf{\Lambda}^\alpha_{n-1})$$

So that

$$\max_{\mathbf{\Lambda}_n} \log \mu_\alpha(\mathbf{\Lambda}_n) = \max_{\mathbf{\Lambda}_n} \log \psi_{n,n-1}(\mathbf{\Lambda}_n, \mathbf{\Lambda}^\alpha_{n-1}) + \log \mu_\alpha(\mathbf{\Lambda}^\alpha_{n-1}) = \max_{\mathbf{\Lambda}_n} \log \psi_{n,n-1}(\mathbf{\Lambda}_n, \mathbf{\Lambda}^\alpha_{n-1})$$

Identical reasoning applies for the backward pass quantities $\mathbf{\Lambda}^\beta_{n+1}$ which may similarly be recursively computed starting from time $N$:

$$\mathbf{\Lambda}_n^\alpha = \mathrm{argmax}_{\mathbf{\Lambda}_n} \log \psi_{n,n-1}(\mathbf{\Lambda}_n, \mathbf{\Lambda}^\alpha_{n-1})$$

$$\mathbf{\Lambda}_n^\beta = \mathrm{argmax}_{\mathbf{\Lambda}_n} \log \psi_{n,n+1}(\mathbf{\Lambda}_n, \mathbf{\Lambda}^\beta_{n+1})$$

□

Analogously, if one were to imagine a similar argument on the latent points $z^t$, which are not explicitly variables in our formulation, one would find on our likelihood 17 that

$$= \mathbb{P}((\mathbf{x}^{n-1}, \mathbf{x}^n, \mathbf{x}^{n+1}), (z^{n-1}, z^n, z^{n+1})) = \int_{\mathbf{x}^{j \notin [n,n-1,n+1]}, \mathbf{z}^{j \notin [n,n-1,n+1]}} \left[ \prod_{i=1}^{N} \mathbb{P}(\mathbf{x}^i \mid z^i) \right] \prod_{i=1}^{N-1} \mathbb{P}(z^i, z^{i+1}) d\mathbf{x}^j dz^j$$

$$= \mu_\alpha(z^{n-1}) \mathbb{P}(z^{n-1}, z^n) \mathbb{P}(\mathbf{x}^n \mid z^n) \mathbb{P}(z^n, z^{n+1}) \mu_\beta(z^{n+1})$$

bioRxiv preprint doi: https://doi.org/10.1101/2025.02.14.638351; this version posted February 19, 2025. The copyright holder for this preprint (which was not certified by peer review) is the author/funder, who has granted bioRxiv a license to display the preprint in perpetuity. It is made available under aCC-BY 4.0 International license.

So that the maximization is expressed

$$\max_{z^{t-1},z^t,z^{t+1}} \log \mu_\alpha(z^{n-1})\mathbb{P}(z^{n-1},z^n)\mathbb{P}(\mathbf{x}^n \mid z^n)\mathbb{P}(z^n,z^{n+1})\mu_\beta(z^{n+1})$$

$$\geq \max_{z^{t-1}} \log \mu_\alpha(z^{n-1}) + \max_{z^n} \log \mathbb{P}(z^{\alpha,n-1},z^n)\mathbb{P}(\mathbf{x}^n \mid z^n)\mathbb{P}(z^n,z^{\beta,n+1}) + \max_{z^{n+1}} \log \mu_\beta(z^{n+1})$$

So that given $z^{\alpha,n-1}$ and $z^{\beta,n+1}$ as forward-backward variables one optimizes $\max_{z^n} \log \mathbb{P}(z^{\alpha,n-1},z^n)\mathbb{P}(\mathbf{x}^n \mid z^n)\mathbb{P}(z^n,z^{\beta,n+1})$ alone, implying that $\mathbb{P}(z^{\alpha,n-1},z^n) := \mathbf{T}^{(n-1,n)}$, $\mathbb{P}(z^n,z^{\beta,n+1}) := \mathbf{T}^{(n,n+1)}$, and $\mathbb{P}(\mathbf{x}^n \mid z^n) := \mathbf{\Lambda}_n$ are optimized given $\mathbf{\Lambda}_{n-1}^\alpha$ and $\mathbf{\Lambda}_{n+1}^\alpha$ fixed. This similarly implies the step:

$$\mathbf{\Lambda}_n^\gamma \leftarrow \min_{\mathbf{\Lambda}_n^\gamma=\mathbf{Q}_n^\gamma \operatorname{diag}(1/\boldsymbol{g}_n^\gamma),\mathbf{T}^{(i,i+1)}} \langle \mathbf{C}^{(n-1,n)}, \mathbf{\Lambda}_{n-1}^\alpha \mathbf{T}^{(n-1,n)} \mathbf{\Lambda}_n^{\gamma T}\rangle_F + \langle \mathbf{C}^{(n,n+1)}, \mathbf{\Lambda}_n^\gamma \mathbf{T}^{(n,n+1)} \mathbf{\Lambda}_{n+1}^{\beta,T}\rangle_F$$

Although a MAP estimate for $\mathbf{\Lambda}_n$ is distinct from the solution in the most likely total sequence $\mathbf{\Lambda}_n \in \vec{\mathbf{\Lambda}}^* = (\mathbf{\Lambda}_1, ..., \mathbf{\Lambda}_n, ..., \mathbf{\Lambda}_N)^*$, one can still find an optimal set of transition matrices $\{\mathbf{T}^{(n,n+1)}\}_{n=1}^{N-1}$ to link them. In particular, given the smoothed matrices $\{\mathbf{\Lambda}_n\}_{n=1}^N$, one may connect them in a transport chain directly by performing a final optimization for the optimal transition between each pair of smoothed clusters.

$$\min_{(\mathbf{T}^{(i,i+1)}\in\Pi(\boldsymbol{g}_i,\boldsymbol{g}_{i+1}))_{i=1}^{N-1}} \sum_{t=1}^{N-1} \langle \mathbf{T}^{(t,t+1)}, \mathbf{\Lambda}_t^T \mathbf{C}^{(t,t+1)} \mathbf{\Lambda}_{t+1}\rangle_F \tag{20}$$

$$= \sum_{t=1}^{N-1} \min_{\mathbf{T}^{(t,t+1)}\in\Pi(\boldsymbol{g}_t,\boldsymbol{g}_{t+1})} \langle \mathbf{T}^{(t,t+1)}, \mathbf{\Lambda}_t^T \mathbf{C}^{(t,t+1)} \mathbf{\Lambda}_{t+1}\rangle_F \tag{21}$$

Thus, by the decoupling of the $\mathbf{T}$ matrices given $\mathbf{\Lambda}$ fixed, this constitutes a simple pairwise OT transport problem for $\mathbf{T}^{(n,n+1)} \in \mathbb{R}_+^{r_n \times r_{n+1}}$ given $\mathbf{\Lambda}_n, \mathbf{\Lambda}_{n+1}$ fixed:

$$\min_{\mathbf{T}^{(n,n+1)}\in\Pi(\boldsymbol{g}_{Q_n},\boldsymbol{g}_{Q_{n+1}})} \langle \mathbf{T}^{(n,n+1)}, \mathbf{\Lambda}_n^T \mathbf{C}^{(n,n+1)} \mathbf{\Lambda}_{n+1}\rangle_F$$

**Algorithm 2** $\alpha$-Pass

Base case: boundary condition for first clustering pair
$\boldsymbol{Q}_1, \boldsymbol{Q}_2 \leftarrow \min_{\boldsymbol{Q}_1,\boldsymbol{Q}_2,\mathbf{T}^{(1,2)}} \langle \mathbf{C}^{(1,2)}, \boldsymbol{Q}_1 \operatorname{diag}(1/\boldsymbol{g}_{\boldsymbol{Q}_1})\mathbf{T}^{(1,2)} \operatorname{diag}(1/\boldsymbol{g}_{\boldsymbol{Q}_2})\boldsymbol{Q}_2^T\rangle_F$
$n \leftarrow 2$
$\boldsymbol{Q}_n \leftarrow \boldsymbol{Q}_2$
**while** $n < N$ **do**
  $\mathbf{\Lambda}_n \leftarrow \boldsymbol{Q}_n \operatorname{diag}(1/\boldsymbol{g}_{\boldsymbol{Q}_n})$ ▷ Memoize variable present in next term of sequence
  $\boldsymbol{Q}_{n+1} \leftarrow \min_{\boldsymbol{Q}_{n+1},\mathbf{T}^{(n,n+1)}} \langle \mathbf{C}^{(n,n+1)}, \mathbf{\Lambda}_n \mathbf{T}^{(n,n+1)} \operatorname{diag}(1/\boldsymbol{g}_{\boldsymbol{Q}_{n+1}})\boldsymbol{Q}_{n+1}^T\rangle_F$
  $n \leftarrow n+1$
  $\boldsymbol{Q}_n \leftarrow \boldsymbol{Q}_{n+1}$
**end while**
**return** $\boldsymbol{Q}_{1:N-1}^\alpha$

### S4.2 Low-rank factorization of products of cost matrices

The only input to Algorithm 1 are the pairwise distance matrices $\mathbf{C}^{(i,i+1)} \in \mathbb{R}^{n_i \times n_{i+1}}$ which capture the dissimilarity between a pair of datasets $\mathbf{X}^{(i)}$ and $\mathbf{X}^{(i+1)}$. However, when $n_i \times n_{i+1}$ is a prohibitively large value, it becomes necessary to take a low-rank factorization of the distance matrix itself. Algorithms to do so have been investigated in [14], and applied to OT cost matrices in [30, 31, 32]. In applications involving multi-modal data, such as spatial-transcriptomics, it is often effective to geometrically average the cost matrices $\{\mathbf{A}^{(i)}\}_{i=1}^K$ as $\mathbf{F}^K = \odot|_{i=1}^K \mathbf{A}^{(i)}$ [10]. In S4.2, following previous works which offer the factorization when one takes the Hadamard product of a low-rank

bioRxiv preprint doi: https://doi.org/10.1101/2025.02.14.638351; this version posted February 19, 2025. The copyright holder for this preprint (which was not certified by peer review) is the author/funder, who has granted bioRxiv a license to display the preprint in perpetuity. It is made available under aCC-BY 4.0 International license.

**Algorithm 3** $\beta$-Pass

Base case: boundary condition for last clustering pair
$\boldsymbol{Q}_N, \boldsymbol{Q}_{N-1} \leftarrow \min_{\boldsymbol{Q}_{N-1},\boldsymbol{Q}_N,\mathbf{T}^{(N-1,N)}} \langle \mathbf{C}^{(N-1,N)}, \boldsymbol{Q}_{N-1}\,\mathrm{diag}(1/\boldsymbol{g}_{\boldsymbol{Q}_{N-1}})\mathbf{T}^{(N-1,N)}\,\mathrm{diag}(1/\boldsymbol{g}_{\boldsymbol{Q}_N})\boldsymbol{Q}_N^T\rangle_F$
$n \leftarrow N-1$
$\boldsymbol{Q}_n \leftarrow \boldsymbol{Q}_{N-1}$
**while** $n > 1$ **do**
  $\boldsymbol{\Lambda}_n \leftarrow \boldsymbol{Q}_n\,\mathrm{diag}(1/\boldsymbol{g}_{\boldsymbol{Q}_n})$ ▷ Memoize variable present in previous term of sequence
  $\boldsymbol{Q}_{n-1} \leftarrow \min_{\boldsymbol{Q}_{n-1},\mathbf{T}^{(n-1,n)}} \langle \mathbf{C}^{(n-1,n)}, \mathrm{diag}(1/\boldsymbol{g}_{\boldsymbol{Q}_{n-1}})\boldsymbol{Q}_{n-1}\mathbf{T}^{(n,n+1)}\boldsymbol{\Lambda}_n^T\rangle_F$
  $n \leftarrow n-1$
  $\boldsymbol{Q}_n \leftarrow \boldsymbol{Q}_{n-1}$
**end while**
**return** $\boldsymbol{Q}^{\beta}_{2:N}$

matrix with itself [32], we prove Proposition 2 to generalize this to the case one has two distinct low-rank matrices $\mathbf{X} = \mathbf{X}_1\mathbf{X}_2^T \in \mathbb{R}^{n\times n}$ and $\mathbf{Y} = \mathbf{Y}_1\mathbf{Y}_2^T \in \mathbb{R}^{n\times n}$ of potentially distinct ranks $d_1, d_2$ for a factorization of $\mathbf{X}\odot\mathbf{Y}$.

$$\mathbf{X}\odot\mathbf{Y} = \mathbf{M}_1\mathbf{M}_2^T := \left(\sum_{i=1}^n \mathbf{e}_i\,\mathrm{vec}\left(\mathbf{x}_i^1\mathbf{y}_i^{(1)T}\right)^T\right)\left(\sum_{j=1}^n \mathbf{e}_j\,\mathrm{vec}\left(\mathbf{x}_j^{(2)}\mathbf{y}_j^{(2)T}\right)^T\right)^T \tag{22}$$

Where $\mathbf{X}_1 = \sum_i \mathbf{e}_i\mathbf{x}_i^{(1)T}$, $\mathbf{X}_2 = \sum_j \mathbf{e}_j\mathbf{x}_j^{(2)T}$, and likewise $\mathbf{Y}_1, \mathbf{Y}_2$. Thus, taking $\mathbf{M}_1\mathbf{M}_2^T = \mathbf{A}^1\odot\mathbf{A}^{(2)}$ as a base case and continuing inductively, one can geometrically average factored distance matrices $\{\mathbf{A}^{(i)}\}_{i=1}^K$ as $\mathbf{F}^K = \odot\,|_{i=1}^K\,\mathbf{A}^{(i)}$.

**Proposition 2.** *Suppose we want to compute* $\mathbf{X}\odot\mathbf{Y}$ *where* $\mathbf{X}$, $\mathbf{Y} \in \mathbb{R}^{n\times n}$ *admit low-rank decompositions of the form* $\mathbf{X} = \mathbf{X}_1\mathbf{X}_2^T$ *and* $\mathbf{Y} = \mathbf{Y}_1\mathbf{Y}_2^T$ *where* $\mathbf{X}_1 \in \mathbb{R}^{n\times d_1}$, $\mathbf{X}_2 \in \mathbb{R}^{m\times d_1}$ *and* $\mathbf{Y}_1 \in \mathbb{R}^{n\times d_2}$, $\mathbf{Y}_2 \in \mathbb{R}^{m\times d_2}$. *Suppose we denote:*

$$\mathbf{X}_1 = \sum_i \mathbf{e}_i\mathbf{x}_i^{(1)T}, \qquad \mathbf{X}_2 = \sum_j \mathbf{e}_j\mathbf{x}_j^{(2)T}, \qquad \mathbf{Y}_1 = \sum_i \mathbf{e}_i\mathbf{y}_i^{(1)T}, \qquad \mathbf{Y}_2 = \sum_j \mathbf{e}_j\mathbf{y}_j^{(2)T}$$

*Then one may use the low-rank factorizations of* $\mathbf{X}$ *and* $\mathbf{Y}$ *to factorize their Hadamard product into matrices of dimension* $\mathbb{R}^{n\times d_1d_2}$

$$\mathbf{X}\odot\mathbf{Y} = \left(\sum_{i=1}^n \mathbf{e}_i\,\mathrm{vec}\left(\mathbf{x}_i^1\mathbf{y}_i^{(1)T}\right)^T\right)\left(\sum_{j=1}^n \mathbf{e}_j\,\mathrm{vec}\left(\mathbf{x}_j^{(2)}\mathbf{y}_j^{(2)T}\right)^T\right)^T := \mathbf{M}_1\mathbf{M}_2^T$$

*Proof.*

$$\mathbf{X}\odot\mathbf{Y} = \mathbf{X}_1\mathbf{X}_2^T\odot\mathbf{Y}_1\mathbf{Y}_2^T = \left(\sum_{i=1}^n \mathbf{e}_i\mathbf{x}_i^{(1)T}\left(\sum_{j=1}^n \mathbf{e}_j\mathbf{x}_j^{(2)T}\right)^T\right)\odot\left(\sum_{i=1}^n \mathbf{e}_i\mathbf{y}_i^{(1)T}\left(\sum_{j=1}^n \mathbf{e}_j\mathbf{y}_j^{(2)T}\right)^T\right)$$

$$= \left(\sum_{i=1}^n\sum_{j=1}^n \mathbf{e}_i\mathbf{x}_i^{(1)T}\mathbf{x}_j^{(2)}\mathbf{e}_j^T\right)\odot\left(\sum_{i=1}^n\sum_{j=1}^n \mathbf{e}_i\mathbf{y}_i^{(1)T}\mathbf{y}_j^{(2)}\mathbf{e}_j^T\right) = \left(\sum_{i=1}^n\sum_{j=1}^n \mathbf{x}_i^{(1)T}\mathbf{x}_j^{(2)}\mathbf{y}_i^{(1)T}\mathbf{y}_j^{(2)}\mathbf{e}_i\mathbf{e}_j^T\right)$$

$$= \sum_{i=1}^n\sum_{j=1}^n \mathrm{tr}\left[\mathbf{x}_j^{(2)T}\mathbf{x}_i^1\mathbf{y}_i^{(1)T}\mathbf{y}_j^{(2)}\right]\mathbf{e}_i\mathbf{e}_j^T = \sum_{i=1}^n\sum_{j=1}^n \mathrm{tr}\left[\mathbf{x}_i^1\mathbf{y}_i^{(1)T}\mathbf{y}_j^{(2)}\mathbf{x}_j^{(2)T}\right]\mathbf{e}_i\mathbf{e}_j^T$$

$$= \sum_{i=1}^n\sum_{j=1}^n \langle\mathbf{x}_i^1\mathbf{y}_i^{(1)T}, \mathbf{x}_j^{(2)}\mathbf{y}_j^{(2)T}\rangle_F\mathbf{e}_i\mathbf{e}_j^T = \sum_{i=1}^n\sum_{j=1}^n \mathbf{e}_i\,\mathrm{vec}\left(\mathbf{x}_i^1\mathbf{y}_i^{(1)T}\right)^T\mathrm{vec}\left(\mathbf{x}_j^{(2)}\mathbf{y}_j^{(2)T}\right)\mathbf{e}_j^T$$

$$= \left(\sum_{i=1}^n \mathbf{e}_i\,\mathrm{vec}\left(\mathbf{x}_i^1\mathbf{y}_i^{(1)T}\right)^T\right)\left(\sum_{j=1}^n \mathbf{e}_j\,\mathrm{vec}\left(\mathbf{x}_j^{(2)}\mathbf{y}_j^{(2)T}\right)^T\right)^T := \mathbf{M}_1\mathbf{M}_2^T$$

□

bioRxiv preprint doi: https://doi.org/10.1101/2025.02.14.638351; this version posted February 19, 2025. The copyright holder for this preprint (which was not certified by peer review) is the author/funder, who has granted bioRxiv a license to display the preprint in perpetuity. It is made available under aCC-BY 4.0 International license.

## S5 Clustering from HM-OT output

Suppose we have a time series $\mathbf{X} = \mathbf{X}^1, \ldots, \mathbf{X}^N$, and HM-OT output: a temporal clustering $\mathbf{Q}, \mathbf{T}$

$$\mathbf{Q}, \mathbf{T} = \{\mathbf{Q}_t\}_{t=1}^N, \{\mathbf{T}^{(t,t+1)}\}_{t=1}^{N-1}.$$

### S5.1 Max-likelihood co-clustering from HM-OT output

The simplest way to extract a hard clustering of each timepoint using $\mathbf{Q}, \mathbf{T}$ is through the $\mathbf{Q}_t$ matrices, each of which is $n_t \times r_t$ in shape. For each $t = 1, \ldots, N$, assign each spot $i \in [n_t]$ to a cluster label $1, \ldots, r_t$ by taking the argmax over each row. For each $t \in [N]$, this corresponds to clustering function

$$f_t^{\max} : \mathbf{X}^t \to [r_t], \quad x_i \mapsto \text{argmax}_{h \in [r_t]} [\mathbf{Q}_t]_{ih},$$

and clustering $\mathcal{C}^{\max,(t)} = \{\mathcal{C}_1^{\max,(t)}, \ldots, \mathcal{C}_{r_t}^{\max,(t)}\}$ given by

$$\mathcal{C}_h^{\max,(t)} = \{x_i \in \mathbf{X}^t : f_t^{\max}(x_i) = h\}.$$

This hard clustering uses the joint probabilities $[\mathbf{Q}_t]_{ih} \equiv \mathbb{P}(x_i^t, z_h^t)$. We call this *max-likelihood clustering* of $\mathbf{X}$ using $\mathbf{Q}, \mathbf{T}$.

It may also be of interest to instead use the corresponding matrices $\mathbf{\Lambda}_t$ of conditional probabilities for the same purpose. Recall that

$$\mathbf{\Lambda}_t = \mathbf{Q}_t \text{diag}(1/\boldsymbol{g}_t), \quad \boldsymbol{g}_t := \mathbf{Q}_t^T \mathbf{1}_{n_t}.$$

From these, clustering function

$$\tilde{f}_t^{\max} : \mathbf{X}^t \to [r_t], \quad x_i \mapsto \text{argmax}_{h \in [r_t]} [\mathbf{\Lambda}_t]_{ih},$$

and corresponding clustering $\tilde{\mathcal{C}}^{\max,(t)} = \{\tilde{\mathcal{C}}_1^{\max,(t)}, \ldots, \tilde{\mathcal{C}}_{r_t}^{\max,(t)}\}$ given by

$$\tilde{\mathcal{C}}_h^{\max,(t)} = \{x_i \in \mathbf{X}^t : \tilde{f}_t^{\max}(x_i) = h\}.$$

As this hard clustering uses the conditional probabilities $[\mathbf{\Lambda}_t]_{ih} \equiv \mathbb{P}(x_i^t \mid z_h^t)$, which play the role of emission probabilities in the HMM interpretation of $\mathbf{Q}, \mathbf{T}$, we call this *emission max-likelihood* clustering of $\mathbf{X}$ using $\mathbf{Q}, \mathbf{T}$.

### S5.2 Reference joint clustering from HM-OT output

There is an alternative clustering making use of the latent coupling matrices $\mathbf{T}$ and a reference timepoint $t_* \in [N]$. All timepoints are then clustered relative to the set of labels $[r_{t_*}]$ at timepoint $t_*$.

This method of clustering relies on the fact that, together $\mathbf{Q}, \mathbf{T}$ define a joint distribution between any two timepoints (not necessarily consecutive) that factors through a composition of latent coupling matrices. Without loss of generality, suppose $s, t \in [N]$ with $s < t$. Define latent coupling matrix $\mathbf{T}^{(s,t)}$ by

$$\mathbf{T}^{(s,t)} = \mathbf{T}^{(s,s+1)} \text{diag}(1/\boldsymbol{g}_{s+1}) \mathbf{T}^{(s+1,s+2)} \text{diag}(1/\boldsymbol{g}_{s+2}) \ldots \text{diag}(1/\boldsymbol{g}_{t-1}) \mathbf{T}^{(t-1,t)}, \tag{23}$$

and define the joint distribution of timepoints $s$ and $t$ from $\mathbf{Q}, \mathbf{T}$ as:

$$\mathbf{P}^{(s,t)} = \mathbf{Q}_s \text{diag}(1/\boldsymbol{g}_s) \mathbf{T}^{(s,t)} \text{diag}(1/\boldsymbol{g}_t) \mathbf{Q}_t^T. \tag{24}$$

We then define clusterings at each timepoint as follows. Starting with the reference timepoint $t_*$, we use max-likelihood clustering (described just above) to assign its labels:

$$f_{t_*}^{\text{ref}(t_*)} : \mathbf{X}^{(t_*)} \to [r_{t_*}], \quad x_i \mapsto \text{argmax}_{h \in [r_{t_*}]} [\mathbf{Q}_t]_{ih} \tag{25}$$

However, for all other timepoints $s$, we use the couplings $\mathbf{P}^{s,t_*}$ and $\mathbf{P}^{t_*,s}$ (depending on whether $s < t_*$) to assign cluster labels. Each of these couplings is used to form a map $\varphi^{(s,t_*)} : \mathbf{X}^{(s)} \to \mathbf{X}^{(t_*)}$, after which labels are assigned to $\mathbf{X}^{(s)}$ by composing with $f_{t_*}^{\text{ref}(t_*)}$ in (25). Concretely,

$$\varphi^{(s,t_*)} : \mathbf{X}^{(s)} \to \mathbf{X}^{(t_*)} := \begin{cases} \text{argmax}_{j \in [n_{t_*}]} [\mathbf{P}^{(s,t_*)}]_{ij} & \text{if } s < t_* \\ \text{argmax}_{i \in [n_{t_*}]} [\mathbf{P}^{(t_*,s)}]_{ij} & \text{if } s > t_*, \end{cases}$$

bioRxiv preprint doi: https://doi.org/10.1101/2025.02.14.638351; this version posted February 19, 2025. The copyright holder for this preprint (which was not certified by peer review) is the author/funder, who has granted bioRxiv a license to display the preprint in perpetuity. It is made available under aCC-BY 4.0 International license.

and we define the reference clustering functions at other timepoints $s$:

$$f_s^{\mathrm{ref}(t_*)} : \mathbf{X}^{(s)} \to [r_{t_*}], \quad f_s^{\mathrm{ref}(t_*)} := f_{t_*}^{\mathrm{ref}(t_*)} \circ \varphi^{(s,t_*)}.$$

Clustering function $f_s^{\mathrm{ref}(t_*)}$ yields a *reference clustering of* $\mathbf{X}^{(s)}$*, relative to timepoint* $t_*$:

$$\mathcal{C}^{\mathrm{ref}(t_*),(s)} = \{\mathcal{C}_1^{\mathrm{ref}(t_*),(s)}, \dots, \mathcal{C}_{r_{t_*}}^{\mathrm{ref}(t_*),(s)}\}, \quad \mathcal{C}_h^{\mathrm{ref}(t_*),(s)} := \{x_i \in \mathbf{X}^{(s)} : f_s^{\mathrm{ref}(t_*)}(x_i) = h\}.$$

# S6 Metrics

## S6.1 Metrics for Differentiation Maps

**Pointwise Mutual-Information (PMI) for Differentiation Maps.** Another measure of the quality of a differentiation map is the deviation between the probability of a transition between cell types $a$ to $b$ and the probability of the same transition under the independence model. Specifically, given the joint distribution $\mathbf{T}^{(s,t)}$, defined in 5, and marginal distributions $\boldsymbol{g}_s$, $\boldsymbol{g}_t$ between any pair of timepoints $s$, $t$, we define the deviation using the *pointwise mutual information*, an information-theoretic metrics widely used in machine-learning [15]:

$$\mathrm{PMI}(a,b) = \log \frac{\mathbb{P}_{s,t}(a,b)}{\mathbb{P}_s(a)\mathbb{P}_t(b)} = \log \frac{\mathbf{T}_{a,b}^{(s,t)}}{(\boldsymbol{g}_s)_a(\boldsymbol{g}_t)_b}.$$

The normalized PMI (NPMI) scales PMI to be in $[-1,+1]$: $+1$ is perfect correspondence, $-1$ is for never co-occurring, and $0$ is for independence.

# S7 Experimental Details

## S7.1 Synthetic Examples

### S7.1.1 2-timepoint toy model of cell-differentiation

We illustrate the advantages of `HM-OT` on a toy model for single-cell differentiation whose dynamics are driven by a "Waddington potential" $\Phi : \mathbb{R}^g \to \mathbb{R}$. In the Waddington framework [33, 38], the velocity with which cells differentiate depends only on their location in expression-space, descending according to the gradient of the Waddington potential: $\dot{\mathbf{x}} = -\nabla\Phi(\mathbf{x}^t)$, where $\mathbf{x}^t = (\mathbf{x}_1^t, \dots, \mathbf{x}_g^t)$ a vector of transcript expression values for some cell at time $t$. For our illustrative example, we simulate cells having $g = 2$ genes, and use a gradient field $-\nabla\Phi$ defined piecewise in each quadrant of $\mathbb{R}^2$, described explicitly in Equation 26 and shown in Fig. S1**a**). We evaluate whether `HM-OT` can cluster points by quadrant when only given an unlabeled timepoint of the dynamics at a later time. We illustrate the distinction between clusters output by `HM-OT`, and those obtained from standard single time-point clustering methods, such as $k$-means. In (Fig. S1**b**), we observe that `HM-OT` (i.e. low-rank OT as a sub-case of `HM-OT` for 2 time points) correctly identifies the initial condition of the four clusters. Single timepoint clustering methods such as $k$-means are unable to identify such structure and cluster the dataset arbitrarily, only clustering on structure intrinsic to the dataset itself. `HM-OT` and low-rank OT are instead able to cluster by an external field (e.g. the Waddington gradient) acting on the data by using temporal information.

$$-\nabla\Phi(\mathbf{z}_t) = \begin{cases} \begin{pmatrix} 2 & 2 \end{pmatrix}^T & \text{if} \quad \mathbf{z}_t \succ \begin{pmatrix} 0 & 0 \end{pmatrix}^T \\ \begin{pmatrix} -2, 2 \end{pmatrix}^T & \text{if} \quad \mathbf{z}_{t,1} > 0, \mathbf{z}_{t,0} < 0 \\ \begin{pmatrix} 2, -2 \end{pmatrix}^T & \text{if} \quad \mathbf{z}_{t,1} < 0, \mathbf{z}_{t,0} > 0 \\ \begin{pmatrix} -2, 2 \end{pmatrix}^T & \text{if} \quad \mathbf{z}_t \prec \begin{pmatrix} 0 & 0 \end{pmatrix}^T \end{cases} \tag{26}$$

Comparing against $K$-means, one finds the AMI against the ground-truth initial condition for the dynamics, `HM-OT` achieves AMI $= 0.873$ and $K$-means achieves AMI $= 0.393$. This highlights the advantage of low-rank optimal transport for clustering on the external field a dataset is subject to, as opposed to the intrinsic structure within it alone.

### S7.1.2 3-timepoint moving Gaussian-mixtures

To demonstrate the value of multiple ($\geq 3$) timepoints in joint-clustering, we compare a latent pairwise optimal transport method (FRLC) to `HM-OT`on a time series dataset with three timepoints $t_1, t_2, t_3$ (Fig. S1**c**). Timepoint $t_1$ is sampled

bioRxiv preprint doi: https://doi.org/10.1101/2025.02.14.638351; this version posted February 19, 2025. The copyright holder for this preprint (which was not certified by peer review) is the author/funder, who has granted bioRxiv a license to display the preprint in perpetuity. It is made available under aCC-BY 4.0 International license.

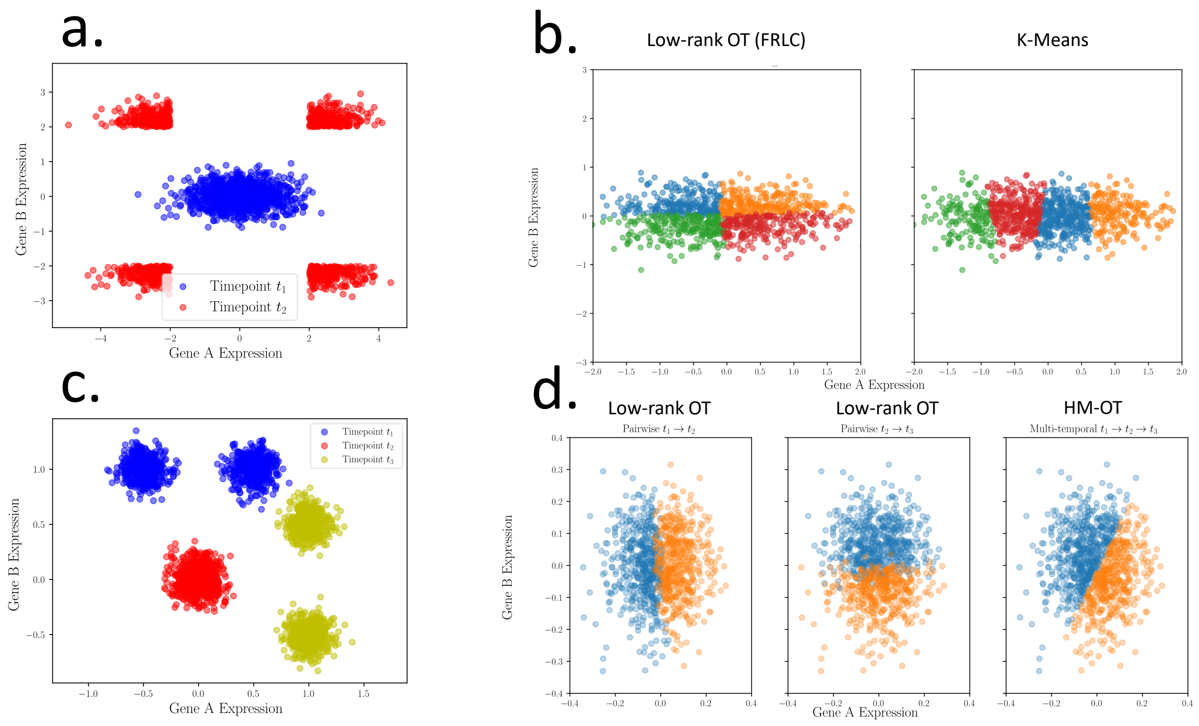


**Figure S1: `HM-OT` on a synthetic example. a.** An example of data from two timepoints with a vector field splitting on four quadrants – the initial condition of $t_1$ determines which of the four clusters the cell differentiates into. **b.** Pairwise clustering using `HM-OT` (2 timepoints), and clustering using $k$-means. **c.** A three-timepoint synthetic example colored according to clusters which minimize respective pairwise or ternary losses. **d.** (Left) Clustering at time $t_2$ obtained by a pairwise alignment of $t_1$ and $t_2$; (Middle) The clustering at time $t_2$ obtained by a pairwise alignment of $t_2$ and $t_3$; (Right) Clustering at $t_2$ obtained via `HM-OT` alignment of all three timepoints.

bioRxiv preprint doi: https://doi.org/10.1101/2025.02.14.638351; this version posted February 19, 2025. The copyright holder for this preprint (which was not certified by peer review) is the author/funder, who has granted bioRxiv a license to display the preprint in perpetuity. It is made available under aCC-BY 4.0 International license.

from a mixture of two bivariate Gaussians with means $\boldsymbol{\mu}_1^{\pm} := (\pm\epsilon, 1)$; timepoint $t_2$ is sampled from a single bivariate, centered Gaussian; and timepoint $t_3$ is sampled from a mixture of two bivariate Gaussians with means $\boldsymbol{\mu}_3^{\pm} := (\pm\epsilon, 0)$. All Gaussians in this example are isotropic, with covariance matrix given by $\sigma^2$ times the identity. Pairwise latent optimal transport identifies cost-minimizing clusters at $t_2$ for each pairwise direction ($t_1 \to t_2$ and $t_2 \to t_3$). The resulting clusters in each case (Fig. S1**d**) have orthogonal boundaries, reflecting the relative positions of $t_1$ or $t_3$ to $t_2$, rather than a consistent differentiation polarity across all three timepoints. HM-OT identifies clusters minimizing the total transport cost $t_1 \to t_2 \to t_3$, and identifies a hyperplane separating progenitor-like and descendant-like cells at $t_2$ using all three time-points. Whereas pairwise optimal transport minimizes according to first-order velocities, HM-OT captures second order information on trajectories, such as curvature or momentum.

## S7.2 Zebrafish embryogenesis, spatial dataset

### S7.2.1 Preprocessing and Hyperparameter Selection

We demonstrate the extension of HM-OT to Zebrafish spatial transcriptomics, using the Stereo-Seq dataset of [21] across 3.3hpf, 5.25hpf, 10hpf, 12hpf, 18hpf, and 24hpf (where hpf denotes hours post fertilization). We use `scanpy` to load the datasets. To generate pairwise costs we take the intersection of common genes between each pair of timepoints to generate a joint AnnData object. Given this joint AnnData, containing all transcriptomic features at both timepoints, we perform a normalization with `scanpy.pp.normalize_total` and add log pseudocounts with `scanpy.pp.log1p`. We project the transcript vectors onto the first 60 principle components of the joint dataset $\mathbf{U}\mathbf{U}^T\mathbf{X}^{(i)}$, computed using `scanpy.pp.pca`. The pairwise costs $\mathbf{C}^{(i,i+1)}$ are computed using these components. In Table S3 we list the hyperparameters used on the spatial Zebrafish dataset. $\alpha > 0$ implies we use a spatial Gromov-Wasserstein term in the objective, to distinguish it from the Wasserstein-only (single-cell) case. We use a Geodesic cost matrix for this GW-term and factorize it with an SVD, as the method takes a distance matrix as input in low-rank form.

### S7.2.2 Differential Expression Analysis for Notochord and Adaxial

The somite and adaxial cell types are approximately divided between HM-OT types (13) ($N = 333$ spots) and (15) ($N = 395$ spots), where (13) is predicted by HM-OT to transition to notochord and (15) is predicted to transition to somite (Fig. 2**a**). Somites are mesodermal pre-muscular cells, and adaxial cells differentiate within the somites to muscle fibers. The notochord, meanwhile, is a rod-like structure which supports the neural tube and is surrounded by adaxial cells [24, 5]. Interestingly, the (13) cell type is more central and flanked by type (15) on both sides–recapitulating how the notochord is a central rod flanked by both somite and adaxial cells [24, 5]. We perform differential expression using a T-test with Benjamini-Hochberg correction. The "adaxial" cell type of [21] has top 5 genes *acta1a* ($Z = 20.68$, for $\alpha$-actin in skeletal muscle), *myl10* ($Z = 19.69$, a myosin muscle protein), *hspb1* and *hsp90aa1.1* ($Z = 19.45$ and $= 11.26$, both heat shock factors), and *chrd* ($Z = 10.87$). Our cell type (15) has 4 of the same genes among the top 5 genes *acta1a* ($Z = 10.61$), *myl10* ($Z = 10.17$), *hspb1* ($Z = 9.96$), *hsp90aa1.1* ($Z = 8.30$), but has *ripply1* ($Z = 6.60$) as the fifth most represented instead of *chrd*, which is no longer among even the top 10 differentially expressed in cell type (15). Chordin is a key notochord marker, and *ripply1* is a gene involved in somite segmentation which also is expressed in the notochord type [16]. None of the top 10 genes in this identified type are muscular, with the expression of the skeletal muscle proteins in adaxial substantially diminished with *acta1a* ($Z = 4.53$) and *myl10* ($Z = 4.76$) substantially less expressed. This recapitulates the established co-localization of the distinct adaxial-somite and notochord cell types [24, 5].

### S7.2.3 Further discussion of NPMI values between 10hpf and 18hpf

Both the HM-OT cell types and [21] suggest yolk-syncytial has $\mathrm{NPMI} = 0.312$ through the graph. Both maps erroneously suggest differentiation occurring from the yolk-syncytial layer – this is partially due to the balanced OT constraint which requires all the cells of yolk-syncytial transfer, and perhaps error in the annotation. It is established that ectodermal cells transition to the neural plate and then the nervous system and neural rod [19]. HM-OT suggests an ectodermal layer of cells, labeled "periderm," gives rise a neural plate intermediate which differentiates into the nervous system. The transitions from HM-OT and annotation [21] cell types both suggest that the neural keel, differentiates into a neural plate intermediate and transitions in a second trajectory to neural crest. The neural keel and neural crest are differentiated from the neural plate, and the transition of neural plate to neural crest is supported in the literature [12]. Observing Figure 2c and d, we see cell types (7) and (9) are consistent with the segmentation of the 18hpf neural crest and nervous system in a manner not captured by the 12hpf annotations.

bioRxiv preprint doi: https://doi.org/10.1101/2025.02.14.638351; this version posted February 19, 2025. The copyright holder for this preprint (which was not certified by peer review) is the author/funder, who has granted bioRxiv a license to display the preprint in perpetuity. It is made available under aCC-BY 4.0 International license.

**Table S1:** NPMI values of HM-OT cell types on developmental zebrafish dataset of [21].

| | Anterior Neural Keel | Notochord | Paraxial Mesoderm, Neural Keel | Periderm | Posterior Neural Keel | Segmental Plate, Tail Bud | Yolk Syncytial Layer |
|---|---|---|---|---|---|---|---|
| Angioblastic Mesenchymal Cell | -0.917 | -0.943 | -0.905 | -0.921 | -0.891 | -0.969 | 0.517 |
| Erythroid Lineage Cell | -0.973 | -0.942 | -0.976 | -0.979 | -0.935 | 0.52 | -0.969 |
| Forebrain | -0.935 | -0.96 | -0.868 | -0.089 | -0.919 | -0.977 | 0.618 |
| Hatching Gland | -0.991 | -1.0 | -1.0 | -0.992 | -1.0 | -1.0 | 0.488 |
| Immature Eye, Midbrain | -0.93 | -0.95 | -0.882 | 0.23 | -0.919 | -0.967 | 0.194 |
| Nervous System | 0.454 | 0.011 | -0.147 | 0.517 | 0.288 | -0.926 | -0.898 |
| Neural Crest | 0.17 | -0.737 | 0.813 | 0.174 | 0.114 | -0.954 | -0.775 |
| Neural Crest, Otic Vesicle | 0.37 | -0.611 | -0.081 | 0.402 | 0.262 | -0.924 | -0.859 |
| Notochord | -0.966 | 0.665 | -0.969 | -0.976 | -0.857 | 0.033 | -0.985 |
| Pronephros | -0.939 | -0.82 | -0.947 | -0.944 | -0.886 | 0.213 | -0.96 |
| Segmental Plate, Tail Bud | -0.702 | 0.252 | -0.749 | -0.722 | -0.651 | -0.032 | -0.892 |
| Somite | -0.96 | -0.264 | -0.962 | -0.975 | -0.837 | 0.497 | -0.986 |
| Yolk Syncytial Layer | -0.989 | -1.0 | -1.0 | -0.99 | -1.0 | -1.0 | 0.312 |
| Yolk Syncytial Layer, Periderm | -0.985 | -0.987 | -0.987 | -0.986 | -0.981 | -0.984 | 0.38 |

**Table S2:** NPMI values of annotated cell types on developmental zebrafish dataset of [21].

| | Anterior Neural Keel | Notochord | Paraxial Mesoderm, Neural Keel | Periderm | Posterior Neural Keel | Segmental Plate, Tail Bud | Yolk Syncytial Layer |
|---|---|---|---|---|---|---|---|
| Angioblastic Mesenchymal Cell | 0.486 | 0.216 | -0.912 | -0.939 | 0.448 | -0.957 | -0.069 |
| Erythroid Lineage Cell | -0.964 | -0.911 | -0.98 | -0.957 | -0.957 | 0.52 | -0.932 |
| Forebrain | -0.786 | -0.834 | -0.887 | 0.214 | -0.851 | -0.963 | 0.501 |
| Hatching Gland | -0.98 | -0.987 | -0.988 | -0.983 | -0.978 | -0.991 | 0.488 |
| Immature Eye, Midbrain | -0.823 | -0.724 | -0.836 | -0.832 | -0.84 | -0.934 | 0.309 |
| Nervous System | 0.147 | -0.708 | 0.541 | 0.394 | -0.674 | -0.971 | -0.864 |
| Neural Crest | 0.145 | -0.352 | 0.428 | 0.294 | -0.031 | -0.967 | 0.028 |
| Neural Crest, Otic Vesicle | 0.395 | 0.174 | -0.709 | -0.706 | 0.384 | -0.947 | -0.058 |
| Notochord | -0.895 | -0.107 | -0.963 | -0.934 | -0.851 | 0.61 | -0.442 |
| Pronephros | -0.913 | -0.716 | -0.954 | -0.864 | -0.914 | -0.931 | 0.293 |
| Segmental Plate, Tail Bud | -0.915 | -0.723 | -0.956 | -0.867 | -0.915 | -0.934 | 0.306 |
| Somite | -0.742 | 0.79 | -0.943 | -0.957 | -0.662 | -0.965 | -0.824 |
| Yolk Syncytial Layer | -0.988 | -0.99 | -1.0 | -0.99 | -1.0 | -0.991 | 0.312 |
| Yolk Syncytial Layer, Periderm | -0.989 | -0.991 | -0.991 | -0.991 | -0.986 | -0.992 | 0.38 |

### S7.2.4 Validation of unsupervised differentiation map in minimizing transcriptional distance to *unseen* costs.

Using HM-OT we infer the differentiation map either with the annotated clusters or the unsupervised clusters HM-OT can additionally learn. To validate the biological accuracy of these unsupervised clusters and their differentiation map, we compute pairwise transcriptional distances between stages separated by greater than one timepoint, HM-OT has no access to as it only uses pairwise distances between adjacent timepoints. A reasonable differentiation map, under compositions of its different stages, should align ancestral and descendant clusters at time $i$ and those at $j$ for $j > i + 1$ with least transcriptional distance. This is demonstrated in Figure **??**, where we show that using the unsupervised clusters the composition of the differentiation map between all timepoints up to 24hpf has lower average transcriptional distance than the differentiation map using annotated clusters: the improvement in the TD ranges from an average of 6.24 transcripts/spot at 18hpf to near zero at 3hpf, which is fully undifferentiated and distant from all differentiated 24hpf clusters.

**Table S3:** HM-OT model Hyperparameters

| Hyperparameter | Description | Value |
|---|---|---|
| $\alpha$; $(1-\alpha)$ | Weight to Gromov-Wasserstein; to Wasserstein | 0.01; 0.99 |
| $\tau_{out}$ | Regularization of outer marginals deviating from $\boldsymbol{a}$ or $\boldsymbol{b}$ | 1 |
| $\tau_{in}$ | Mirror-descent regularization of inner marginal at step $\boldsymbol{g}_Q^k$ deviating from $\boldsymbol{g}_Q^{k-1}$ | $1 \times 10^{-7}$ |
| $\gamma$ | Mirror-descent step-size | 30 |

**Table S4:** Hyperparameters of HM-OT on developmental zebrafish dataset of [21].

### S7.2.5 Unsupervised Cluster Loss on Standard OT-Costs

As a simple baseline, we also evaluate whether the inferred unsupervised clusters from Problem 2, where $(\mathbf{Q}, \mathbf{T})$ are jointly minimized, achieve a lower value of the Wasserstein and Gromov-Wasserstein loss than the supervised clusters from Problem 3 where $\mathbf{T}$ is minimized and $\mathbf{Q}$ is fixed from annotation [21]. We demonstrate, for a Fused GW objective in Table S5 that, unsurprisingly, the cost is lower when $\mathbf{Q}$ is also learned.

bioRxiv preprint doi: https://doi.org/10.1101/2025.02.14.638351; this version posted February 19, 2025. The copyright holder for this preprint (which was not certified by peer review) is the author/funder, who has granted bioRxiv a license to display the preprint in perpetuity. It is made available under aCC-BY 4.0 International license.

| Dataset | | Annot W | HM-OT W | Annot GW | HM-OT GW |
|---|---|---|---|---|---|
| hpf3 to hpf5 | 3,5,10 joint | 3.439 | **3.408** | 5.952 | **5.518** |
| hpf5 to hpf10 | 3,5,10 joint | 3.531 | **3.461** | 6.776 | **5.952** |
| hpf5 to hpf10 | 5,10,12 joint | 3.529 | **3.500** | 10.562 | **6.969** |
| hpf10 to hpf12 | 5,10,12 joint | 2.425 | **2.354** | 6.844 | **5.021** |
| hpf10 to hpf12 | 10,12,18 joint | 2.484 | **2.389** | 6.697 | **4.906** |
| hpf12 to hpf18 | 10,12,18 joint | 2.827 | **2.727** | 4.531 | **3.916** |
| hpf12 to hpf18 | 12,18,24 joint | 3.154 | **3.042** | 5.013 | **4.619** |
| hpf18 to hpf24 | 12,18,24 joint | 3.127 | **2.913** | 4.363 | **3.480** |

**Table S5:** Performance of HM-OT clusters (Algorithm 1) against annotation clusters in minimizing the Wasserstein and Gromov-Wasserstein distance across four joint alignments of time triples $(t_1, t_2, t_3)$. For each clustering, the optimal transport cost for the associated clusters is computed and shown – for this spatial dataset, a larger contribution has been given to the GW-term.

## S7.3 Mouse Embryonic development

### S7.3.1 Preprocessing and Hyperparameter Selection

We follow the exact procedure for preprocessing outlined in S7.2.1, and offer the hyperparameters used for generating the mouse embryo differentiation map (Figure 3) in S7.

### S7.3.2 Evaluating Scalability

To evaluate scalability, we ran HM-OT on all time pairs of the MOSTA Stereo-Seq dataset [3]. We evaluate the performance on low-rank pairwise alignment (setting `max_iter=60`, as used in the experiments) for direct comparison with `moscot`. Unlike `moscot`, HM-OT can run on all timepoints of the dataset, up to E16.5 which has $N = 121767$ spots. Moreover, the scaling of runtime with dataset size is minimal (Figure S8a) – this is due to the FRLC algorithm [11] which scales low-rank OT using Sinkhorn [4], as opposed to previous works which relied on the less efficient Dykstra subroutine [30, 8, 20] which is used in `moscot` [17]. We also show the improvement in space complexity of HM-OT in using a low-rank coupling factorization over storing the quadratic full-rank matrix (Figure S8b).

| | E9.5_10.5 | E10.5_11.5 | E11.5_12.5 | E12.5_13.5 | E13.5_14.5 | E14.5_15.5 | E15.5_16.5 |
|---|---|---|---|---|---|---|---|
| moscot (solve only) | 13.950 | 25.647 | 24.347 | N/A | N/A | N/A | N/A |
| HM-OT | 8.985 | 9.077 | 9.105 | 9.141 | 9.251 | 9.304 | 9.311 |

**Table S6:** Runtimes of `moscot` and HM-OT across different time-points in seconds.

| Hyperparameter | Description | Value |
|---|---|---|
| $\alpha$; $(1-\alpha)$ | Weight to Gromov-Wasserstein; to Wasserstein | $10^{-4}$; $1 - 10^{-4}$ |
| $\tau_{out}$ | Regularization of outer marginals deviating from $\boldsymbol{a}$ or $\boldsymbol{b}$ | 5 |
| $\tau_{in}$ | Mirror-descent regularization of inner marginal at step $\boldsymbol{g}_Q^k$ deviating from $\boldsymbol{g}_Q^{k-1}$ | $1 \times 10^{-5}$ |
| $\gamma$ | Mirror-descent step-size | 40 |

**Table S7:** Hyperparameters of HM-OT on MOSTA [3] Stereo-Seq mouse organogenesis dataset.

# S8 Supplemental Figures

bioRxiv preprint doi: https://doi.org/10.1101/2025.02.14.638351; this version posted February 19, 2025. The copyright holder for this preprint (which was not certified by peer review) is the author/funder, who has granted bioRxiv a license to display the preprint in perpetuity. It is made available under aCC-BY 4.0 International license.

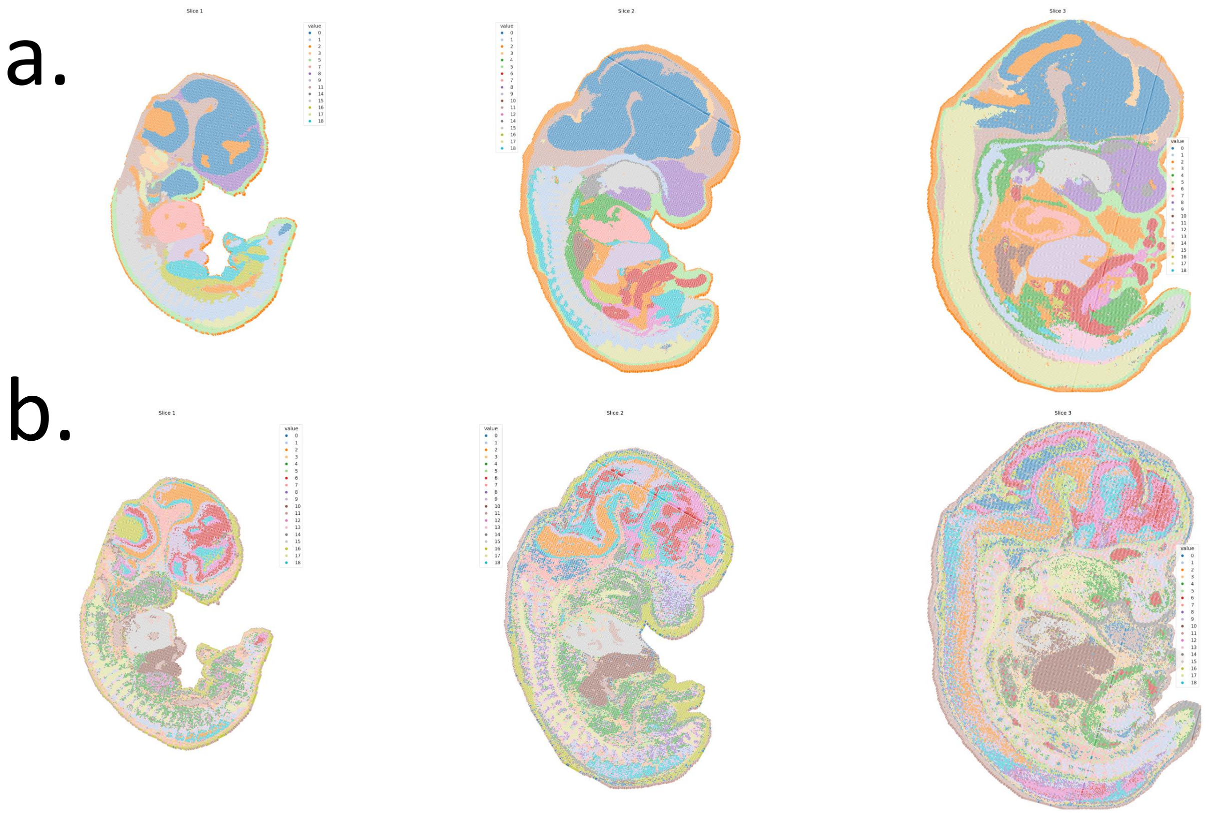


**Figure S2:** Ancestral clustering on the MOSTA mouse organogenesis dataset of [3] with reference index 5 on **a.** the [3] annotations and the `HM-OT` differentiation map for linking them, **b.** `HM-OT` unsupervised cell types. As the reference index is the final timepoint, this corresponds to "pulling back" differentiated cell types to their progenitors.

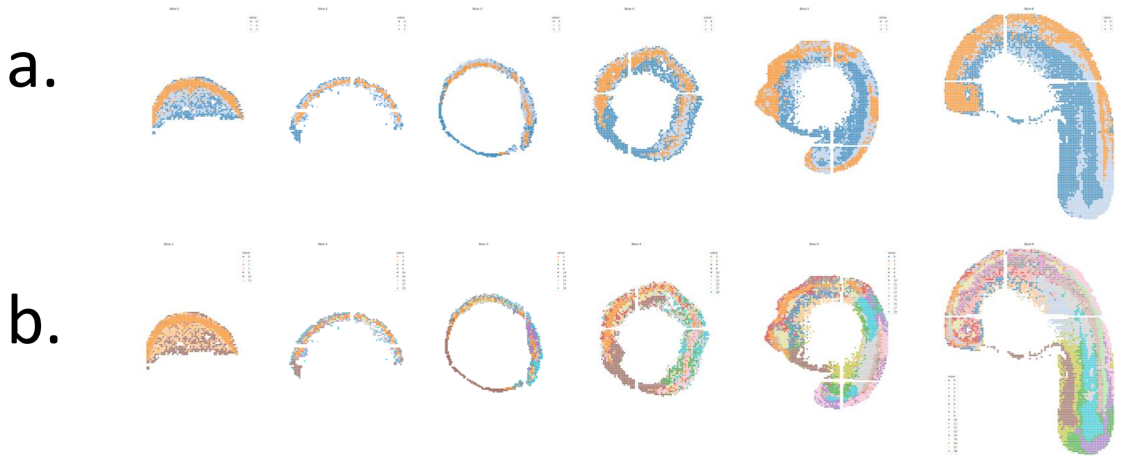


**Figure S3:** Ancestral clustering on the zebrafish dataset of [21] with fully-unsupervised cell types inferred by `HM-OT` with **a.** reference index zero corresponding to mapping progenitor cells into future descendant locations, **b.** ancestral clustering with reference index 5, corresponding to “pulling back” differentiated cell types to their progenitors.

bioRxiv preprint doi: https://doi.org/10.1101/2025.02.14.638351; this version posted February 19, 2025. The copyright holder for this preprint (which was not certified by peer review) is the author/funder, who has granted bioRxiv a license to display the preprint in perpetuity. It is made available under aCC-BY 4.0 International license.

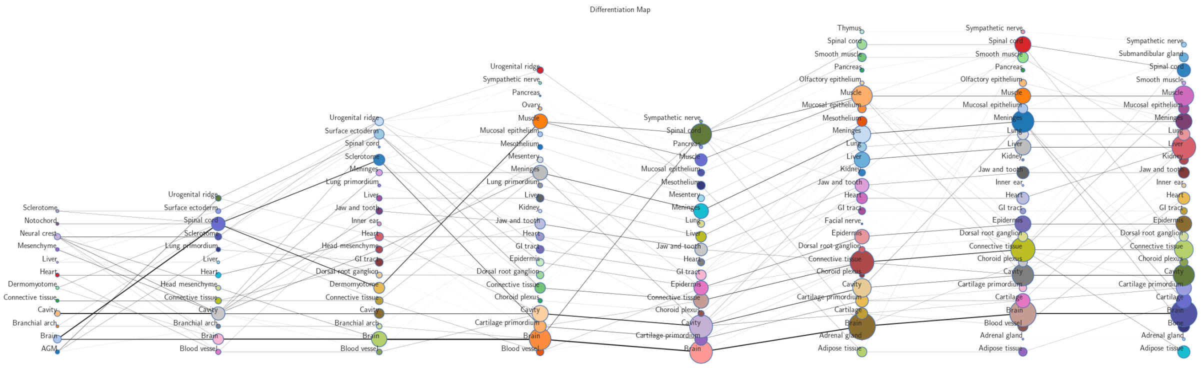


**Figure S4:** Full differentiation map of Stereo-Seq mouse organogenesis (MOSTA) dataset [3] from timepoints E9.5-E16.5 generated using `HM-OT`.

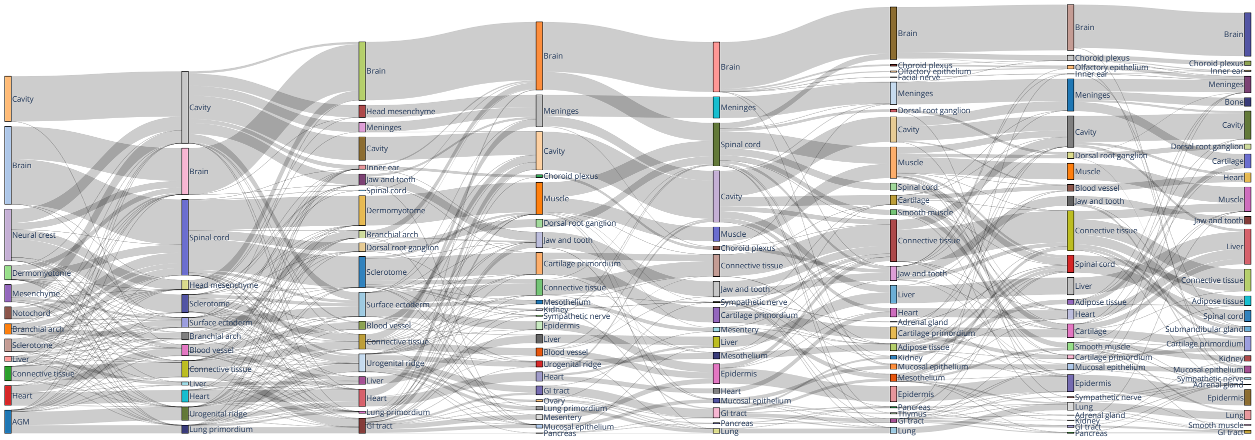

**Figure S5:** A Sankey plot corresponding to the differentiation map in Figure S4, from E9.5 to E16.5.

bioRxiv preprint doi: https://doi.org/10.1101/2025.02.14.638351; this version posted February 19, 2025. The copyright holder for this preprint (which was not certified by peer review) is the author/funder, who has granted bioRxiv a license to display the preprint in perpetuity. It is made available under aCC-BY 4.0 International license.

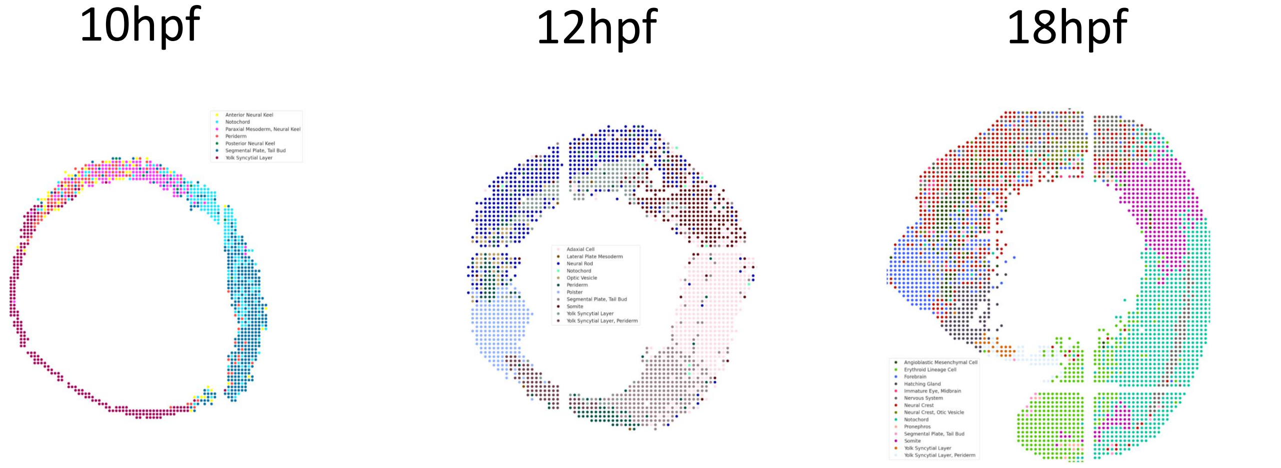


**Figure S6:** Annotated cell types of [21] plotted spatially for the 10hpf, 12hpf, and 18hpf timepoints.

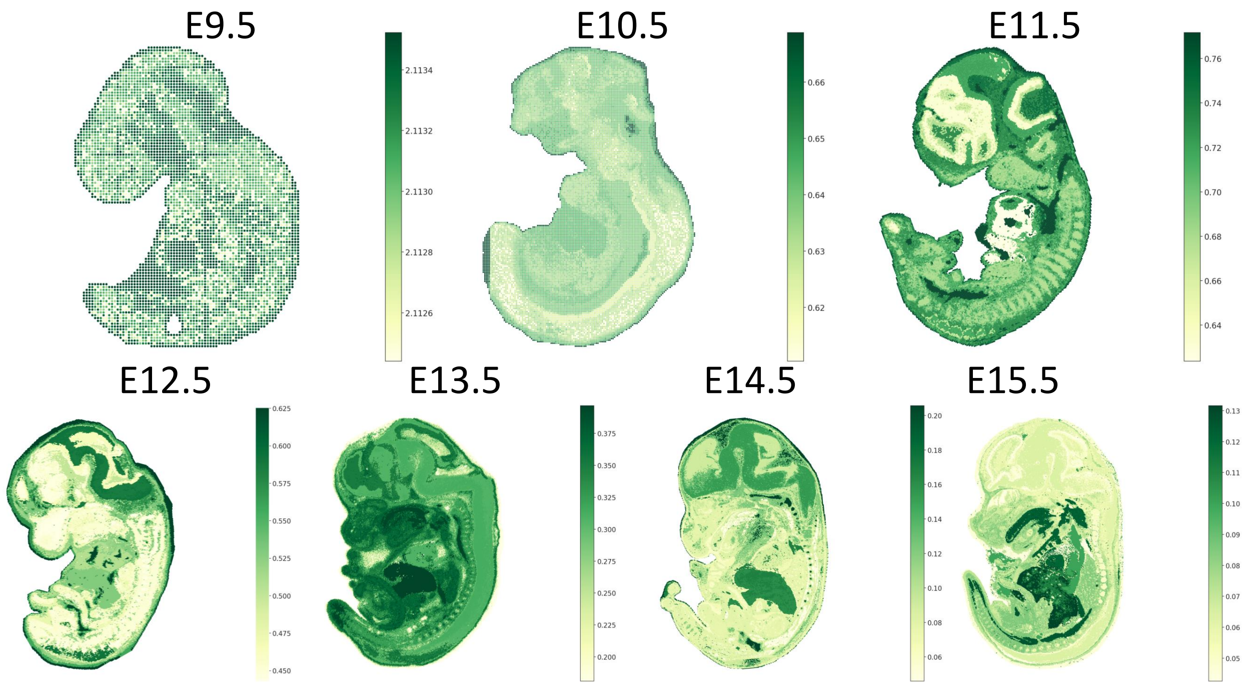


**Figure S7:** Growth-rates on of Stereo-Seq mouse organogenesis (MOSTA) dataset [3] growth-rates ($\xi$) on stages E9.5-15.5 computed in accordance with [10] on the unsupervised `HM-OT` clusters. Growth-rates are scaled to represent the expected number of cells added and sub-couplings computed scalably using the FRLC low-rank optimal transport framework [11].

.

bioRxiv preprint doi: https://doi.org/10.1101/2025.02.14.638351; this version posted February 19, 2025. The copyright holder for this preprint (which was not certified by peer review) is the author/funder, who has granted bioRxiv a license to display the preprint in perpetuity. It is made available under aCC-BY 4.0 International license.

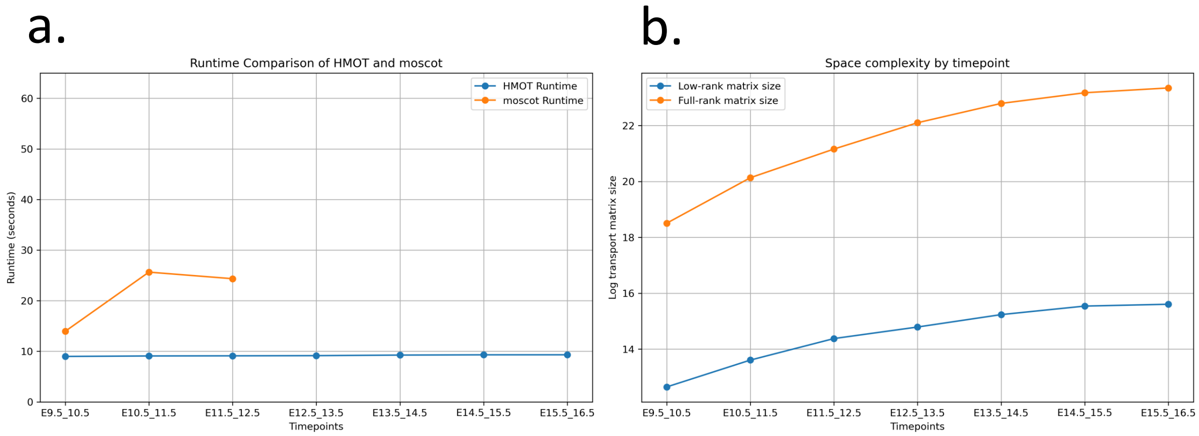


**Figure S8: a.** The runtime of `moscot` (`SpatioTemporalProblem.solve` runtime only) and `HM-OT` across pairwise alignments from E9.5-10.5 to E15.5-16.5. `moscot` fails to run after E11.5-12.5. **b.** The memory-scaling of the low-rank transport matrix solved for by `HM-OT` versus the analogously shaped full-rank matrix.

.

## References

[1] Christopher M. Bishop. *Pattern Recognition and Machine Learning (Information Science and Statistics)*. Springer-Verlag, Berlin, Heidelberg, 2006.

[2] Martin Catala, Marie-Aimée Teillet, and Nicole M. Le Douarin. Organization and development of the tail bud analyzed with the quail-chick chimaera system. *Mechanisms of Development*, 51(1):51–65, May 1995.

[3] Ao Chen, Sha Liao, Mengnan Cheng, Kailong Ma, Liang Wu, Yiwei Lai, Xiaojie Qiu, Jin Yang, Jiangshan Xu, Shijie Hao, et al. Spatiotemporal transcriptomic atlas of mouse organogenesis using dna nanoball-patterned arrays. *Cell*, 185(10):1777–1792, 2022.

[4] Marco Cuturi. Sinkhorn distances: Lightspeed computation of optimal transport. *Advances in Neural Information Processing Systems*, pages 2292–2300, 2013.

[5] Bruno Della Gaspera, Laure Weill, and Christophe Chanoine. Evolution of somite compartmentalization: A view from xenopus. *Frontiers in Cell and Developmental Biology*, 9, January 2022.

[6] Tamal K Dey, Alfred Rossi, and Anastasios Sidiropoulos. Temporal clustering. *arXiv preprint arXiv:1704.05964*, 2017.

[7] Joana Esteves de Lima and Frédéric Relaix. Master regulators of skeletal muscle lineage development and pluripotent stem cells differentiation. *Cell Regeneration*, 10(1), October 2021.

[8] Aden Forrow, Jan-Christian Hütter, Mor Nitzan, Philippe Rigollet, Geoffrey Schiebinger, and Jonathan Weed. Statistical Optimal Transport via Factored Couplings. In Kamalika Chaudhuri and Masashi Sugiyama, editors, *Proceedings of the Twenty-Second International Conference on Artificial Intelligence and Statistics*, volume 89 of *Proceedings of Machine Learning Research*, pages 2454–2465. PMLR, 16–18 Apr 2019.

[9] Mario Ganau, Rahel Zewude, and Michael G. Fehlings. *Functional Anatomy of the Spinal Cord*, page 3–12. Springer International Publishing, December 2018.

[10] Peter Halmos, Xinhao Liu, Julian Gold, Feng Chen, Li Ding, and Benjamin J. Raphael. DeST-OT: Alignment of spatiotemporal transcriptomics data. *Cell Systems*, January 2025.

[11] Peter Halmos, Xinhao Liu, Julian Gold, and Benjamin Raphael. Low-Rank Optimal Transport through Factor Relaxation with Latent Coupling. In *The Thirty-eighth Annual Conference on Neural Information Processing Systems*, 2024.

bioRxiv preprint doi: https://doi.org/10.1101/2025.02.14.638351; this version posted February 19, 2025. The copyright holder for this preprint (which was not certified by peer review) is the author/funder, who has granted bioRxiv a license to display the preprint in perpetuity. It is made available under aCC-BY 4.0 International license.


[12] Xiao Huang and Jean-Pierre Saint-Jeannet. Induction of the neural crest and the opportunities of life on the edge. *Developmental Biology*, 275(1):1–11, November 2004.

[13] Laertis Ikonomou, Michael J. Herriges, Sara L. Lewandowski, Robert Marsland, Carlos Villacorta-Martin, Ignacio S. Caballero, David B. Frank, Reeti M. Sanghrajka, Keri Dame, Maciej M. Kańduła, Julia Hicks-Berthet, Matthew L. Lawton, Constantina Christodoulou, Attila J. Fabian, Eric Kolaczyk, Xaralabos Varelas, Edward E. Morrisey, John M. Shannon, Pankaj Mehta, and Darrell N. Kotton. The in vivo genetic program of murine primordial lung epithelial progenitors. *Nature Communications*, 11(1), January 2020.

[14] Pitor Indyk, Ali Vakilian, Tal Wagner, and David P Woodruff. Sample-optimal low-rank approximation of distance matrices. In Alina Beygelzimer and Daniel Hsu, editors, *Proceedings of the Thirty-Second Conference on Learning Theory*, volume 99 of *Proceedings of Machine Learning Research*, pages 1723–1751. PMLR, 25–28 Jun 2019.

[15] Dan Jurafsky and James H. Martin. *Speech and Language Processing: An Introduction to Natural Language Processing, Computational Linguistics, and Speech Recognition*. Prentice-Hall, 2000.

[16] Akinori Kawamura, Sumito Koshida, Hiroko Hijikata, Akiko Ohbayashi, Hisato Kondoh, and Shinji Takada. Groucho-associated transcriptional repressor ripply1 is required for proper transition from the presomitic mesoderm to somites. *Developmental Cell*, 9(6):735–744, December 2005.

[17] Dominik Klein, Giovanni Palla, Marius Lange, Michal Klein, Zoe Piran, Manuel Gander, Laetitia Meng-Papaxanthos, Michael Sterr, Aimée Bastidas-Ponce, Marta Tarquis-Medina, Heiko Lickert, Mostafa Bakhti, Mor Nitzan, Marco Cuturi, and Fabian J. Theis. Mapping cells through time and space with moscot. *bioRxiv*, 2023.

[18] Elena Kozhemyakina, Andrew B. Lassar, and Elazar Zelzer. A pathway to bone: signaling molecules and transcription factors involved in chondrocyte development and maturation. *Development*, 142(5):817–831, March 2015.

[19] Parvat Kuwar Chhetri and Joe M Das. Neuroanatomy, neural tube development and stages. In *StatPearls*. StatPearls Publishing, Treasure Island (FL), January 2024.

[20] Chi-Heng Lin, Mehdi Azabou, and Eva L Dyer. Making transport more robust and interpretable by moving data through a small number of anchor points. *Proceedings of machine learning research*, 139:6631, 2021.

[21] Chang Liu, Rui Li, Young Li, Xiumei Lin, Kaichen Zhao, Qun Liu, Shuowen Wang, Xueqian Yang, Xuyang Shi, Yuting Ma, Chenyu Pei, Hui Wang, Wendai Bao, Junhou Hui, Tao Yang, Zhicheng Xu, Tingting Lai, Michael Arman Berberoglu, Sunil Kumar Sahu, Miguel A Esteban, Kailong Ma, Guangyi Fan, Yuxiang Li, Shiping Liu, Ao Chen, Xun Xu, Zhiqiang Dong, and Longqi Liu. Spatiotemporal mapping of gene expression landscapes and developmental trajectories during zebrafish embryogenesis. *Dev. Cell*, 57(10):1284–1298.e5, May 2022.

[22] Xinhao Liu, Ron Zeira, and Benjamin J. Raphael. Partial alignment of multislice spatially resolved transcriptomics data. *Genome Research*, 33(7):1124–1132, 2023.

[23] Xiaogao Meng, Guizhong Cui, and Guangdun Peng. Lung development and regeneration: newly defined cell types and progenitor status. *Cell Regeneration*, 12(1), April 2023.

[24] Mai E. Nguyen-Chi, Robert Bryson-Richardson, Carmen Sonntag, Thomas E. Hall, Abigail Gibson, Tamar Sztal, Wendy Chua, Thomas F. Schilling, and Peter D. Currie. Morphogenesis and cell fate determination within the adaxial cell equivalence group of the zebrafish myotome. *PLoS Genetics*, 8(10):e1003014, October 2012.

[25] Chengxiang Qiu, Junyue Cao, Beth K Martin, Tony Li, Ian C Welsh, Sanjay Srivatsan, Xingfan Huang, Diego Calderon, William Stafford Noble, Christine M Disteche, Stephen A. Murray, Malte Spielmann, Cecilia B. Moens, Cole Trapnell, and Jay Shendure. Systematic reconstruction of cellular trajectories across mouse embryogenesis. *Nature genetics*, 54(3):328–341, 2022.

bioRxiv preprint doi: https://doi.org/10.1101/2025.02.14.638351; this version posted February 19, 2025. The copyright holder for this preprint (which was not certified by peer review) is the author/funder, who has granted bioRxiv a license to display the preprint in perpetuity. It is made available under aCC-BY 4.0 International license.


[26] Chengxiang Qiu, Beth K Martin, Ian C Welsh, Riza M Daza, Truc-Mai Le, Xingfan Huang, Eva K Nichols, Megan L Taylor, Olivia Fulton, Diana R O'Day, Anne Roshella Gomes, Saskia Ilcisin, Deng Xinxian Srivatsan, Sanjay, Christine M. Disteche, William Stafford Noble, Nobuhiko Hamazaki, Cecilia B. Moens, David Kimelman, Junyue Cao, Alexander F. Schier, Malte Spielmann, Stephen A. Murray, Cole Trapnell, and Jay Shendure. A single-cell time-lapse of mouse prenatal development from gastrula to birth. *Nature*, 626(8001):1084–1093, 2024.

[27] Ying Qu, Bo Zhou, Wei Yang, Bingchen Han, Yi Yu-Rice, Bowen Gao, Jeffery Johnson, Clive N. Svendsen, Michael R. Freeman, Armando E. Giuliano, Dhruv Sareen, and Xiaojiang Cui. Transcriptome and proteome characterization of surface ectoderm cells differentiated from human ipscs. *Scientific Reports*, 6(1), August 2016.

[28] Wouter Saelens, Robrecht Cannoodt, Helena Todorov, and Yvan Saeys. A comparison of single-cell trajectory inference methods. *Nature biotechnology*, 37(5):547–554, 2019.

[29] Meyer Scetbon and Marco Cuturi. Low-rank optimal transport: Approximation, statistics and debiasing. In Alice H. Oh, Alekh Agarwal, Danielle Belgrave, and Kyunghyun Cho, editors, *Advances in Neural Information Processing Systems*, 2022.

[30] Meyer Scetbon, Marco Cuturi, and Gabriel Peyré. Low-rank sinkhorn factorization. In *International Conference on Machine Learning*, 2021.

[31] Meyer Scetbon, Michal Klein, Giovanni Palla, and Marco Cuturi. Unbalanced low-rank optimal transport solvers, 2023.

[32] Meyer Scetbon, Gabriel Peyré, and Marco Cuturi. Linear-time Gromov Wasserstein Distances using Low Rank Couplings and Costs. In *International Conference on Machine Learning*, pages 19347–19365. PMLR, 2022.

[33] Geoffrey Schiebinger, Jian Shu, Marcin Tabaka, Brian Cleary, Vidya Subramanian, Aryeh Solomon, Joshua Gould, Siyan Liu, Stacie Lin, Peter Berube, et al. Optimal-transport analysis of single-cell gene expression identifies developmental trajectories in reprogramming. *Cell*, 176(4):928–943, 2019.

[34] Corina Schmidt, Mechthild Stoeckelhuber, Iain McKinnell, Reinhard Putz, Bodo Christ, and Ketan Patel. Wnt 6 regulates the epithelialisation process of the segmental plate mesoderm leading to somite formation. *Developmental Biology*, 271(1):198–209, July 2004.

[35] Satomi S. Tanaka and Ryuichi Nishinakamura. Regulation of male sex determination: genital ridge formation and sry activation in mice. *Cellular and Molecular Life Sciences*, 71(24):4781–4802, August 2014.

[36] Vayer Titouan, Nicolas Courty, Romain Tavenard, and Rémi Flamary. Optimal transport for structured data with application on graphs. In *International Conference on Machine Learning*, pages 6275–6284. PMLR, 2019.

[37] Alexander Tong, Jessie Huang, Guy Wolf, David Van Dijk, and Smita Krishnaswamy. TrajectoryNet: A dynamic optimal transport network for modeling cellular dynamics. In Hal Daumé III and Aarti Singh, editors, *Proceedings of the 37th International Conference on Machine Learning*, volume 119 of *Proceedings of Machine Learning Research*, pages 9526–9536. PMLR, 13–18 Jul 2020.

[38] C.H. Waddington. *The Strategy of the Genes*. Routledge, April 2014.

[39] Xiaoyu Wei, Sulei Fu, Hanbo Li, Yang Liu, Shuai Wang, Weimin Feng, Yunzhi Yang, Xiawei Liu, Yan-Yun Zeng, Mengnan Cheng, et al. Single-cell stereo-seq reveals induced progenitor cells involved in axolotl brain regeneration. *Science*, 377(6610):eabp9444, 2022.

[40] Ron Zeira, Max Land, Alexander Strzalkowski, and Benjamin J. Raphael. Alignment and integration of spatial transcriptomics data. *Nature Methods*, 19(5):567–575, 2022.
